# Increased RUNX3 expression mediates tumor‐promoting ability of human breast cancer‐associated fibroblasts

**DOI:** 10.1002/cam4.6421

**Published:** 2023-08-28

**Authors:** Yu Koyama, Hiroya Okazaki, Yang Shi, Yoshihiro Mezawa, Zixu Wang, Mizuki Sakimoto, Akane Ishizuka, Yasuhiko Ito, Takumi Koyama, Yataro Daigo, Atsushi Takano, Yohei Miyagi, Tomoyuki Yokose, Toshinari Yamashita, Keisuke Sugahara, Okio Hino, Liying Yang, Reo Maruyama, Akira Katakura, Takehiro Yasukawa, Akira Orimo

**Affiliations:** ^1^ Department of Oral Pathobiological Science and Surgery Tokyo Dental College Tokyo Japan; ^2^ Department of Pathology and Oncology Juntendo University Faculty of Medicine Tokyo Japan; ^3^ Department of Molecular Pathogenesis Juntendo University Graduate School of Medicine Tokyo Japan; ^4^ Center for Antibody and Vaccine Therapy, Institute of Medical Science, Research Hospital The University of Tokyo Tokyo Japan; ^5^ Department of Medical Oncology and Cancer Center, and Center for Advanced Medicine against Cancer Shiga University of Medical Science Otsu Japan; ^6^ Molecular Pathology and Genetics Division Kanagawa Cancer Center Research Institute Yokohama Japan; ^7^ Department of Pathology Kanagawa Cancer Center Yokohama Japan; ^8^ Department of Breast Surgery and Oncology Kanagawa Cancer Center Yokohama Japan; ^9^ Project for Cancer Epigenomics, Cancer Institute Japanese Foundation for Cancer Research Tokyo Japan; ^10^ Present address: Department of Immunological Diagnosis Juntendo University Graduate School of Medicine Tokyo Japan

**Keywords:** breast cancer, CAFs, cancer‐associated fibroblasts, RUNX3, tumor microenvironment

## Abstract

**Background:**

Cancer‐associated fibroblasts (CAFs) are a major stromal component of human breast cancers and often promote tumor proliferation, progression and malignancy. We previously established an experimental CAF (exp‐CAF) cell line equipped with a potent tumor‐promoting ability. It was generated through prolonged incubation of immortalized human mammary fibroblasts with human breast cancer cells in a tumor xenograft mouse model.

**Results:**

Herein, we found that the exp‐CAFs highly express Runt‐related transcription factor 3 (RUNX3), while counterpart fibroblasts do not. In breast cancer patients, the proportion of RUNX3‐positive stromal fibroblast‐like cells tends to be higher in cancerous regions than in non‐cancerous regions. These findings suggest an association of RUNX3 with CAF characteristics in human breast cancers. To investigate the functional role of RUNX3 in CAFs, the exp‐CAFs with or without shRNA‐directed knockdown of RUNX3 were implanted with breast cancer cells subcutaneously in immunodeficient mice. Comparison of the resulting xenograft tumors revealed that tumor growth was significantly attenuated when RUNX3 expression was suppressed in the fibroblasts. Consistently, Ki‐67 and CD31 immunohistochemical staining of the tumor sections indicated reduction of cancer cell proliferation and microvessel formation in the tumors formed with the RUNX3‐suppressed exp‐CAFs.

**Conclusion:**

These results suggest that increased RUNX3 expression could contribute to the tumor‐promoting ability of CAFs through mediating cancer cell growth and neoangiogenesis in human breast tumors.

## Graphical abstract

Koyama et al. found an elevated expression of Runt‐related transcription factor 3 (RUNX3) in human breast cancer‐associated fibroblasts (CAFs). Data obtained with an experimentally generated CAF line suggest that increased RUNX3 expression could contribute to the tumor‐promoting ability of CAFs through mediating cancer cell growth and neoangiogenesis in human breast tumors.

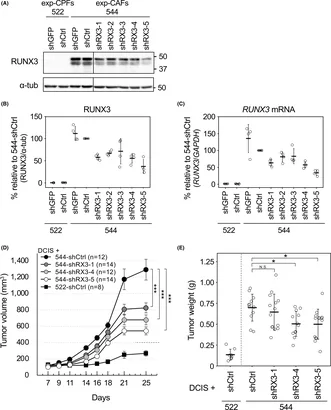

## 1 INTRODUCTION

The Runt‐related transcription factors (RUNX) are crucial regulators of various developmental and cellular processes, such as proliferation, differentiation and cell lineage specification.^1^ They consist of a larger α subunit and a smaller β subunit. In mammals, there are three α subunit proteins, designated RUNX1, RUNX2, and RUNX3. These proteins share a well conserved domain called the Runt domain, which was originally found in *Drosophila runt* gene and mediates their binding to DNA and to the common binding partner, core‐binding factor subunit‐β (CBFβ).^2^ While RUNX proteins play important physiological roles, their dysregulation is linked to tumorigenesis.^3^ Among them, RUNX3 is involved in neurogenesis^4^, ^5^ and T‐cell development.^6^, ^7^ Also, RUNX3 is required for proper differentiation of gastric epithelium.^8^ Expression of RUNX3 is altered in various cancers. Curiously, it is known to act as a tumor suppressor as well as a promoter,^9^, ^10^, ^11^ suggesting that the effect of aberrant RUNX3 expression in cancer is context‐dependent, dependent on cancer cell types and on the tumor characters in which the cancer cells reside. RUNX3 was reported to be involved in transforming growth factor β (TGF‐β)‐induced growth inhibition in gastric cancer cells: upon TGF‐β stimulation, RUNX3 cooperates with Smad proteins to increase transcription of a cyclin‐dependent kinase inhibitor *p21*.^12^ Also reported was that RUNX3 is responsible for transcriptional upregulation of a proapoptotic factor *Bim* in TGF‐β‐induced apoptosis.^13^ Moreover, it was proposed that RNUX3 attenuates Wnt‐signaling by interacting with β‐catenin/T cell factor 4 (TCF4) transcription complexes in intestinal tumorigenesis.^14^ These reports are consistent with the tumor suppressor role of RUNX3. On the other hand, RUNX3 was shown to promote cell proliferation in ovarian cancer cells, indicating that RUNX3 can also play an oncogenic role in cancer cells.^15^ While dysregulation of RUNX3 expression has been reported in various cancer cells and the effect and cause of the dysregulation were extensively studied,^10^ the involvement of RUNX3 in tumor stroma was documented only in a single publication in which RUNX3 was included in the stromal gene set associated with good clinical outcomes of breast carcinomas.^16^

Human breast tumors are composed of cancer cells and nonneoplastic stromal cells. Cancer‐associated fibroblasts (CAFs) are a major component of the latter.^17^ They are activated through reciprocal interaction with cancer cells and have distinct characteristics from fibroblasts in healthy tissue.^18^ CAFs crucially contribute to breast cancer progression by providing growth factors, cytokines, chemokines, and extracellular matrix proteins to tumor microenvironment. We previously demonstrated that breast cancer patient‐derived CAFs highly expressed a typical myofibroblast marker α‐smooth muscle actin (α‐SMA), secreted elevated levels of a proangiogenic chemokine, stromal cell‐derived factor‐1 (SDF‐1, also known as CXCL12) and promoted the growth of cancer cells in a mouse xenograft model.^19^ Subsequently, we have generated CAFs from immortalized human mammary fibroblasts by long‐term incubation with MCF‐7‐ras human breast carcinoma cells in tumors formed under the skin of immunodeficient mice.^20^ The resulting CAFs, designated as experimentally generated CAFs (named exp‐CAF‐2), showed increased α‐SMA expression and tumor‐promoting ability in mouse xenograft tumors, suggesting that the exp‐CAFs recapitulated the key characteristics of primary CAFs isolated from patients with invasive breast carcinomas.^20^ It was proposed that the exp‐CAFs maintain their characteristics through autocrine signaling loops involving SDF‐1 and TGF‐β in cross‐communicating fashion.^20^ Moreover, the exp‐CAFs were shown to induce invasion and metastasis of breast cancer cells,^21^ which further supports an idea that the exp‐CAFs equip with properties of tumor‐promoting CAFs.

To further understand the tumor‐promoting properties of the exp‐CAFs besides SDF‐1 and TGF‐β signaling, we examined their gene expression profile. Curiously, strong induction of RUNX3 expression was detected. In the present study, we therefore investigated the role of aberrant RUNX3 expression in CAFs. We propose that increased RUNX3 expression could contribute to the tumor‐promoting ability of CAFs.

## 2 MATERIALS AND METHODS

### 2.1 Cell culture

Human exp‐CAFs and counterpart fibroblasts (exp‐CPFs) used in this study were established previously.^20^ Briefly, primary normal mammary fibroblasts isolated from reduction mammoplasty tissue were immortalized with human telomerase reverse transcriptase and green fluorescent protein (GFP) and puromycin‐resistance protein were introduced. The resulting cells, called 218TGpp, were used to establish exp‐CAF and exp‐CPF lines. exp‐CAF 544 cells (exp‐CAF‐2) were generated from 218TGpp cells that were injected subcutaneously with MCF‐7‐ras human breast cancer cells into an immunodeficient nude mouse and were incubated for a long‐term (242 days; see details in^20^). exp‐CPF 522 cells went through the same procedure as 544 cells but without MCF‐7‐ras cells. 522 cells, called counterpart fibroblast‐2 cells in^20^, were used as a counterpart fibroblast line. In addition, similar to 544 cells, 881L, 547R, and 542M cells were previously generated through incubation with MCF‐7‐ras cells for 85, 170, and 242 days, respectively. 533 cells were generated similarly to 522 cells. These fibroblasts were cultured in DMEM (Nacalai Tesque 08459‐64) with 10% (v/v) fetal bovine serum (FBS) and penicillin–streptomycin (PS) (Nacalai Tesque 26253‐84). Ductal carcinoma in situ (DCIS) breast cancer cells used in this study are tdTomato‐labeled DCIS.com cells established previously.^21^ They were cultured in DMEM/Ham's F‐12 (Nacalai Tesque 11581‐15) with 5% FBS and PS. 293T cells were cultured in RPMI1640 with 10% FBS and PS.

### 2.2 RNA extraction and reverse transcription‐quantitative PCR

Total RNA was prepared from cultured cells using NucleoSpin RNA (TaKaRa). cDNA was synthesized using PrimeScript RT reagent Kit (TaKaRa) and appropriately diluted. RNA expression levels of target genes were examined with the cDNA samples as templates by real‐time quantitative PCR (qPCR) using Fast SYBR Green Master Mix (Thermo Fisher Scientific) or THUNDERBIRD Next SYBR qPCR Mix (TOYOBO). Expression levels were normalized to *GAPDH*. Primers for qPCR are listed in Table S1.

### 2.3 Western blotting

Total cellular lysates were fractionated by SDS‐PAGE and blotted onto PVDF membranes (Millipore). Target proteins were detected by western blotting using the following primary antibodies: anti‐RUNX3 (R3‐5G4; Santa Cruz, sc101553), anti‐αSMA (Dako, M0851) and anti‐α‐tubulin (Sigma, T5168) antibodies. Secondary antibodies used were horseradish peroxidase conjugates. Protein bands were detected and processed using a ChemiDoc MP instrument and Image Lab ver. 4.1 (Bio‐Rad Laboratories). Moderate adjustment of western blotting images was performed where appropriate.

### 2.4 Immunofluorescence imaging of cultured cells

Cells cultured on glass coverslips were fixed with 4% paraformaldehyde in PBS for 10 min, followed by permeabilization with 0.1% Triton‐X100 in PBS for 5 min. After blocking, the samples were incubated with an anti‐RUNX3 antibody (A‐3) and then Alexa Fluor 568‐conjugated anti‐mouse IgG. Immunofluorescence was viewed using an All‐in‐One Fluorescence Microscope BZ‐X800 (KEYENCE). Acquired images of RUNX3 staining were subjected to haze reduction using software BZ‐X800 Analyzer.

### 2.5 Immunohistochemistry of patient specimens

Formalin‐fixed paraffin‐embedded (FFPE) invasive breast carcinomas were prepared from patients with breast cancer who had received neither preoperative chemotherapy nor hormone therapy. Using an anti‐RUNX3 antibody (R3‐5G4), immunohistochemical staining was performed according to a previous procedure^22^ and the presence of RUNX3 in stroma was evaluated. Each of five different fields in fibroblast‐rich stroma in cancerous and noncancerous regions were captured per slide under a microscope at 40× magnification. Fibroblast‐like cells were distinguished morphologically from tumor cells and other stromal cells, such as leukocytes and vascular endothelial cells. The percentage of RUNX3‐positive fibroblast‐like cells was calculated as the ratio of the number of RUNX3‐positive fibroblast‐like cells to that of all fibroblast‐like cells at each of five fields from cancerous and noncancerous regions and the mean value was respectively obtained from 10 breast cancer patients. For double‐staining of RUNX3 and α‐SMA, after visualization of RUNX3 using 3,3′‐diaminobenzidine (DAB) as chromogen, immunohistochemistry procedure was repeated for α‐SMA using anti‐α‐SMA antibodies (1A4; Agilent Technologies) as primary antibodies and Vulcan Fast Red (Vulcan Fast Red Chromogen kit 2; Biocare Medical) as chromogen with secondary antibodies conjugated with alkaline phosphatase (MACH 2 Double Stain 1; Biocare Medical).

### 2.6 Tissue microarray

Tissue microarrays were constructed using formalin‐fixed tumor specimens dissected from 241 breast cancer patients. After selection of tissue area morphologically by hematoxylin and eosin (HE) staining on each slide, microarray blocks were generated from paraffin embedded tissue cores (diameter 0.6 mm; height 3–4 mm) by tissue microarrayer (Beecher Instruments). Sections were stained with an anti‐RUNX3 antibody (R3‐5G4). Staining was evaluated by researchers who had not been communicated the sample information prior to the evaluation. “Stromal RUNX3‐positive” or “stromal RUNX3‐negative” were defined with a cut‐off rate of 10% positive staining of the total stromal areas.

### 2.7 Gene silencing using short‐hairpin RNA

To achieve gene silencing of *RUNX3*, a lentivirus packaging system was used to introduce short‐hairpin RNA (shRNA) expression constructs into cultured cells. shRNA‐expressing plasmids were generated by inserting shRNA sequences into pLKO1.hygro vector (a gift from Bob Weinberg [Addgene_24150]) using AgeI and EcoRI restriction sites. DNA sequence of the oligonucleotides for shRNA constructs are listed in Table S2. Two shRNA‐expressing plasmids used as controls in this study were generated previously. shRNAs were nonmammalian sequence‐shRNA (shCtrl)^23^ and GFP‐shRNA (shGFP).^20^ Lentivirus particles containing shRNA expression constructs were produced by transfecting 293 T cells with pLKO1.hygro carrying shRNA genes, pCMV‐VSVG and pCMV‐dR8.2dvpr. Then, fibroblasts were infected with the virus particles to integrate shRNA expression constructs into the genome. After infection, 544 and 522 cells were cultured for four days in the medium with hygromycin (25 and 50 μg/mL, respectively) and for another three days in the medium with hygromycin (12.5 and 25 μg/mL, respectively) to eliminate uninfected cells. Then, they were further cultured in the medium without hygromycin for three to six days, after which cells were harvested for downstream analyses and implantation to mice.

### 2.8 Animal experiments

Male NOD/Shi‐*scid*/IL‐2Rγ^null^ (NOG) mice at six weeks of age were purchased from CLEA Japan Inc. (Tokyo). Mice were bred under germ‐free and specific pathogen‐free conditions. For subcutaneous injection to mice, cancer cells and fibroblasts were harvested by trypsinization and washed once with PBS and cell numbers were counted. 0.9 × 10^5^ cancer cells and 2.7 × 10^5^ fibroblasts in 44% Matrigel (Corning) were injected into mice to form xenograft tumors. After injection, the major (*x*) and minor (*y*) axes of tumors were measured at indicated days and tumor volume was calculated with the formula of 4/3*π*(*x*/2)(*y*/2). Tumors were resected 25 days after injection and their weight was measured, after which tumors were fixed with 10% formalin/100 mM sodium phosphate buffer (pH 7.2) and embedded in paraffin. Formalin‐fixed, paraffin‐embedded (FFPE) sections were prepared from the tumors.

### 2.9 Immunohistochemical staining of xenograft tumors and quantitation analyses

FFPE sections were dewaxed with xylene and treated with ethanol. Endogenous peroxidase activity was then eliminated using 0.3% hydrogen peroxide in methanol. Slides were incubated in citric acid buffer solution (pH 6.0) at 121°C for 20 min to retrieve antigens. After blocking, slides were incubated with primary antibodies diluted with Dako REAL Antibody Diluent (Dako). Primary antibodies used are anti‐Ki‐67 (MIB‐1; Dako, M7240) and anti‐CD31 (Abcam, ab28364) antibodies in 1:150 and 1:100 dilution, respectively. Then, slides were incubated with Dako EnVision+ System‐ HRP Labelled Polymer anti‐mouse or ‐rabbit (Dako), followed by DAB staining to visualize the target proteins. Finally, slides were stained with hematoxylin. In addition, HE staining was performed to different serial FFPE sections.

For quantitation analyses, eight fields rich in Ki‐67‐ or CD31‐positive cells were captured in each slide under a microscope at 40× or 20× magnification and the images were analyzed using Image J software as follows. For Ki‐67‐stained specimens, an object classifier was trained to define cells with DAB‐positive and ‐negative nuclei using several captured images. Because cells are occasionally clumped together in clusters, watershed separation was applied so that cells were individually recognized. Also, objects with ≥100 pixels were considered as nuclei. The number of Ki‐67‐positive and ‐negative cells were counted and the ratio of positive cells to positive and negative cells were obtained. For CD31‐stained specimens, an object classifier was trained to define DAB‐positive and ‐negative areas using several captured images. Then, areas surrounded by DAB‐positive cells are considered as microvessels. Because DAB‐positive cells surrounding a vessel are not always continuously present on a thin layer section, Gaussian blur was applied to fill in the gaps between the cells for easier identification of vascular walls. Then, median blur was used at 3.5‐pixel radius to reduce the noise generated by Gaussian blur. Finally, blood vessel areas indicated by CD31‐positive cells were measured (CD31‐positive cells were included in the areas). In addition, the number of blood vessels were counted.

### 2.10 RNA‐sequencing and data processing

**FIGURE 1:**
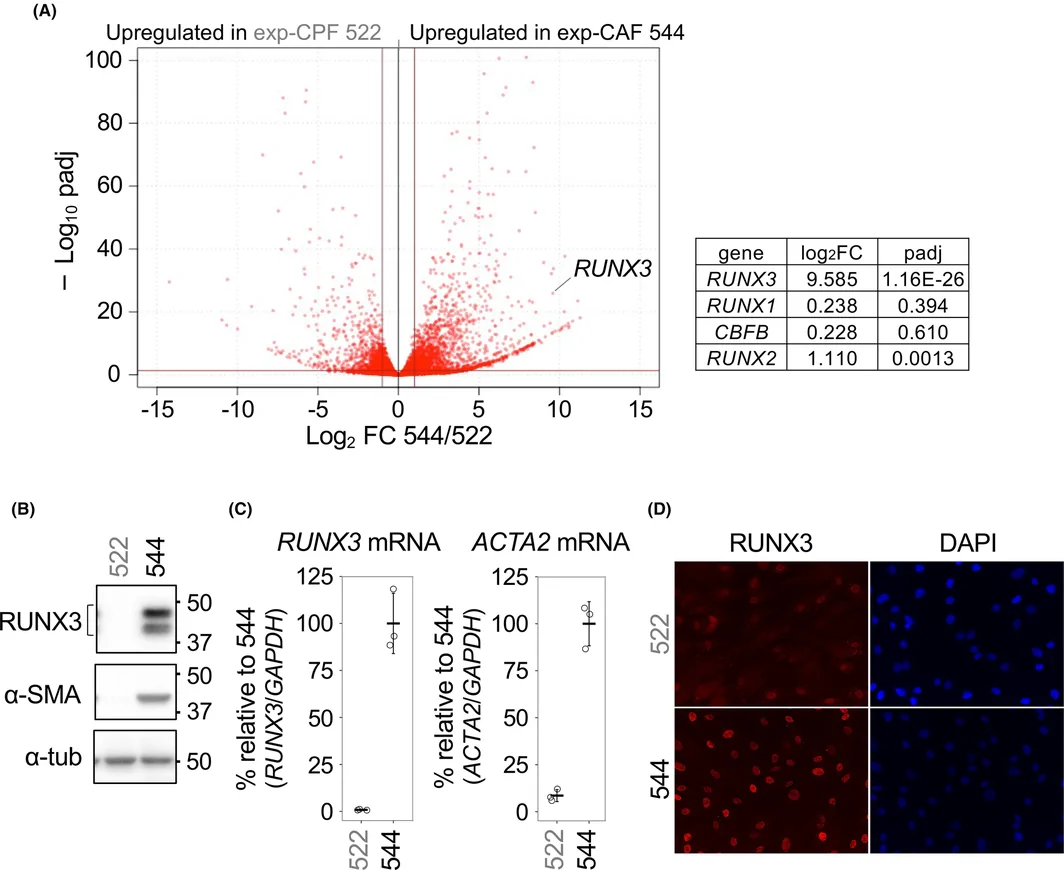
Analysis of RUNX3 expression in experimentally established CAF 544 cells. (A) Volcano plots showing the differences in the global RNA expression between exp‐CAF 544 and exp‐CPF 522 cells. RNA‐seq results with three independent preparations were examined by DESeq2. X‐ and Y‐axes respectively show the log_2_ fold‐change (FC) in 544 cells relative to 522 cells and −log_10_ of the adjusted *p*‐value (padj). Log_2_ FC of ±1 (X‐axis) and padj of 0.05 (Y‐axis) are indicated by brown lines. The dot for RUNX3 is pointed. The table shows the values of Log_2_ FC and padj of the indicated genes. (B) Western blot analysis of RUNX3 and α‐SMA. α‐tubulin (α‐tub) was used as a loading control. (C) RT‐qPCR analysis of *RUNX3* and *ACTA2* mRNAs. Gene expression levels of *RUNX3* and *ACTA2* were normalized against those of *GAPDH*. The normalized levels of *RUNX3*/*GAPDH* and *ACTA2* /*GAPDH* of 544 cells in the three independent preparations were averaged and expressed as 100, and those of all samples were calculated relative to them. The graphs are the average of the results from three independent preparations with SD. (D) Immunofluorescent imaging of RUNX3 in 522 and 544 cells with anti‐RUNX3 antibodies. Nuclei were marked with DAPI.

Total RNA was prepared as above. RNA‐sequencing (RNA‐seq) and data processing were outsourced to Genome‐Lead Co., Ltd (Kagawa, Japan). Briefly, RNA integrity was confirmed using a LabChip GX Touch (PerkinElmer) and RNA Quality Score was ≥8.5 in all samples. mRNA was purified using KAPA mRNA Capture kit (KAPA BIOSYSTEMS) and RNA‐seq libraries were constructed using an MGIEasy RA Directional Library Prep Set (MGI Tech Co., Ltd.). Libraries were sequenced using a DNBSEQ‐G400RS High‐throughput Sequencing Set and FCL PE150 flow cell on a DNABSEQ‐400RS sequencer (MGI Tech Co., Ltd.). For RNA‐seq data presented in Figure 1A, adaptor sequences were removed using TrimGalore (ver. 0.6.6) (https://www.bioinformatics.babraham.ac.uk/projects/trim_galore/). Low‐quality reads (Q < 20) were trimmed and resulting reads shorter than 20 bases were removed using FastqPuri ver. 1.0.^24^ For RNA‐seq data in Figure 5A, similar quality filtering was performed using fastp (ver. 0.6.6) (https://github.com/OpenGene/fastp#fastp). Reads were then aligned against GRCh38.105 human genome using HISAT2 (version 2.2.1)^25^ by RSEM (ver. 1.3.3).^26^ Expected read counts and transcripts per million (TPM) per gene were extracted.

### 2.11 RNA‐seq data analyses

From the list of genes with expected read counts and TPM, lines containing “lnc_RNA”, “5S_rRNA”, “biotype = …”, “#N/A” and “pseudogene” were removed. Genes with 0 counts in all samples were removed. Differential gene expression analysis was performed using Bioconductor package DESeq2 (ver. 1.34.0)^27^ and genes whose read counts were equal to and more than the number of samples were retained. When differentially expressed genes were examined between 522 and 544 cells, RNA‐seq data from three independent RNA samples of each cell line were used (Figure 1A). To analyze the effects of RUNX3 knockdown in 544 cells, RNA‐seq data of six RNA samples from three independent harvests of 544 cells expressing shCtrl and those expressing shGFP and RNA‐seq data of six RNA samples from three independent harvests of 544 cells expressing shRX3‐4 and those expressing shRX3‐5 were compared (Figure 5A). Volcano plots were generated using R (ver. 4.1.3). For heatmap presentation, significantly differentially expressed genes (Log_2_ fold‐change | > 1 and adjusted *p*‐value <0.05) were selected from the DESeq2‐processed data. Hierarchical clustering of the genes was based on Ward's method and Euclidian distance was used for clustering of the samples. Heatmap.2 included in Bioconductor package gplots was used to generate a heatmap using R.

## 3 RESULTS

### 3.1 RUNX3 is highly expressed in the experimentally established CAFs

To gain a deeper understanding of the tumor‐promoting ability of exp‐CAFs, we performed RNA‐seq to compare gene expression profiles of the previously established exp‐CAF‐2 cells and counterpart fibroblast‐2 cells,^20^ which are respectively called exp‐CAF 544 and exp‐CPF 522 cells in this study. exp‐CAF 544 cells were generated from immortalized human mammary fibroblasts that were subjected to long‐term (242 days) exposure to MCF‐7‐ras human breast cancer cells through subcutaneous incubation of the mixture of these cells in immunodeficient nude mice. 544 cells showed tumor‐promoting ability toward MCF‐7‐ras and DCIS breast cancer cells in mouse xenograft models.^20^, ^21^ exp‐CPF 522 cells went through the same procedure as 544 cells without MCF‐7‐ras cells and did not promote tumor growth.^20^, ^21^

RNA‐seq analysis revealed that the expression levels of *RUNX3* were highly upregulated in 544 cells compared to 522 cells (Figure 1A). Among 1936 significantly differentially expressed genes showing more than twofold expression in 544 cells compared to 522 cells, *RUNX3* was ranked within the top 1%. On the o, expression levels of *RUNX1* and *CBFB* had no significant difference between 544 and 522 cells and those of *RUNX2* were only modestly higher in the former (Figure 1A and Figure S1). To reinforce the finding of RUNX3's expression in RNA‐seq, we performed RT‐qPCR and western blotting and confirmed that 544 cells express RUNX3 at both mRNA and protein levels (Figure 1B,C). As reported previously,^20^ while 522 cells show very low expression levels of α‐SMA (gene name: *ACTA2*), 544 cells highly express this protein (Figure 1B,C). Moreover, immunofluorescent imaging indicated the nuclear localization of RUNX3 in 544 cells (Figure 1D), suggesting that RUNX3 could function as a transcription factor to modify the expression of various genes in the fibroblasts. In addition, other exp‐CAF lines also express RUNX3 (Figure S2).

### 3.2 RUNX3 expression is upregulated in stromal fibroblast‐like cells of human breast cancer

**FIGURE 2:**
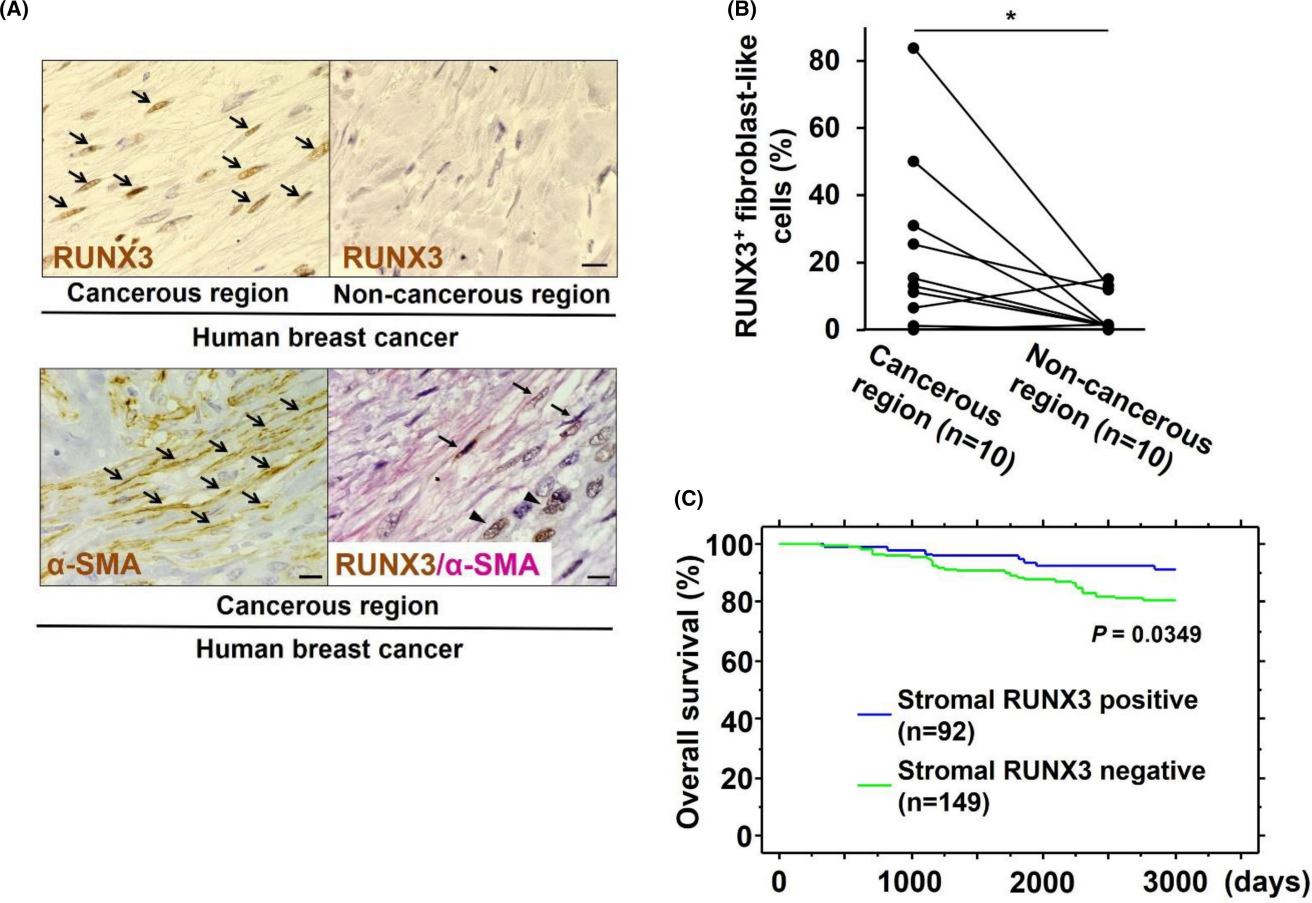
Examination of RUNX3 expression in breast cancer patients. (A) A case of immunohistochemical analysis of human mammary carcinoma sections including cancerous and noncancerous regions. The upper panels are stained with anti‐RUNX3 antibodies, the lower left panel with anti‐α‐SMA antibodies and the lower right panel with both antibodies. RUNX3‐ and α‐SMA‐positive fibroblast‐like cells are shown with arrows in the upper left and lower left panels, respectively. RUNX3 and α‐SMA double‐positive fibroblast‐like cells and RUNX3‐positive cancer cells are indicated with arrows and arrowheads, respectively, in the lower right panel. The upper right panel presents a noncancerous region from the same specimen. Scale bars, 10 μm. (B) Proportions (%) of fibroblast‐like cells positive for RUNX3 staining in cancerous and noncancerous regions of 10 breast cancer patients with α‐SMA‐positive stroma. * (*p* < 0.05) by paired *t*‐test. (C) Kaplan–Meier survival plot of 241 human breast cancer patients based on stromal RUNX3 staining in their tumors. Specimens were categorized to be positive when ≥10% of the stromal region was positive for RUNX3 staining.

To examine whether RUNX3 expression is upregulated in tumor‐associated stroma of breast cancer patients, we performed immunohistochemical staining of patient tumor sections using antibodies against α‐SMA and RUNX3. Ten patient specimens with α‐SMA‐positive myofibroblasts in tumor stroma were analyzed for RUNX3 (Figure 2A and Table S3). Significantly greater proportion of the nuclear RUNX3‐positive fibroblast‐like cells was detected in cancerous regions compared to the patient‐matched noncancerous regions: averaged RUNX3‐positive cell proportion was 23.7% in the former and 4.6% in the latter (Figure 2B). In addition, we found higher *RUNX3* mRNA expression levels in human breast CAFs (Table S4) in a published microarray dataset that was generated with primary cultures of fibroblasts from breast carcinomas and those from the adjacent noncancerous breast tissue from six patients (GSE20086).^28^ These two observations indicate that increased RUNX3 expression is associated with human breast CAFs.

Furthermore, to investigate an association of stromal RUNX3 expression with the prognoses in breast cancer patients, tumor sections prepared from 241 breast cancer patients (Table 1) were stained with an anti‐RUNX3 antibody and subjected to tissue microarray analysis. Patients with positive stromal RUNX3 staining showed a better survival outcome than those with negative RUNX3 staining (Figure 2C; positivity was given when more than 10% of the stromal region analyzed was positive for RUNX3 staining).

**TABLE 1:** Histopathological information of 241 breast cancer patients.

Parameters	Total (*n* = 241)	Stromal RUNX3 positive (*n* = 92)	Stromal RUNX3 negative (*n* = 149)	*p*‐Value
Age (years)				
−50	72	24	48	0.3849
51–	169	68	101	
Grading				
0	37	12	25	0.692 (Grading 0 and 1 vs. 2 and 3)
1	80	31	49	
2	65	22	43	
3	59	27	32	
pT factor				
T1	83	34	49	0.5773
T2‐3	158	58	100	
pN factor				
N0	136	53	83	0.7906
N1‐2	105	39	66	
Luminal				
Luminal A/B	169	71	98	0.0817
Others	72	21	51	
Her2 status				
HER2 type	31	13	18	0.6939
Others	210	79	131	

*Note*: Statistical significance was analyzed by Fisher's exact test.

### 3.3 shRNA‐directed knockdown of RUNX3 attenuates the tumor‐promoting ability of exp‐CAF 544 cells

**FIGURE 3:**
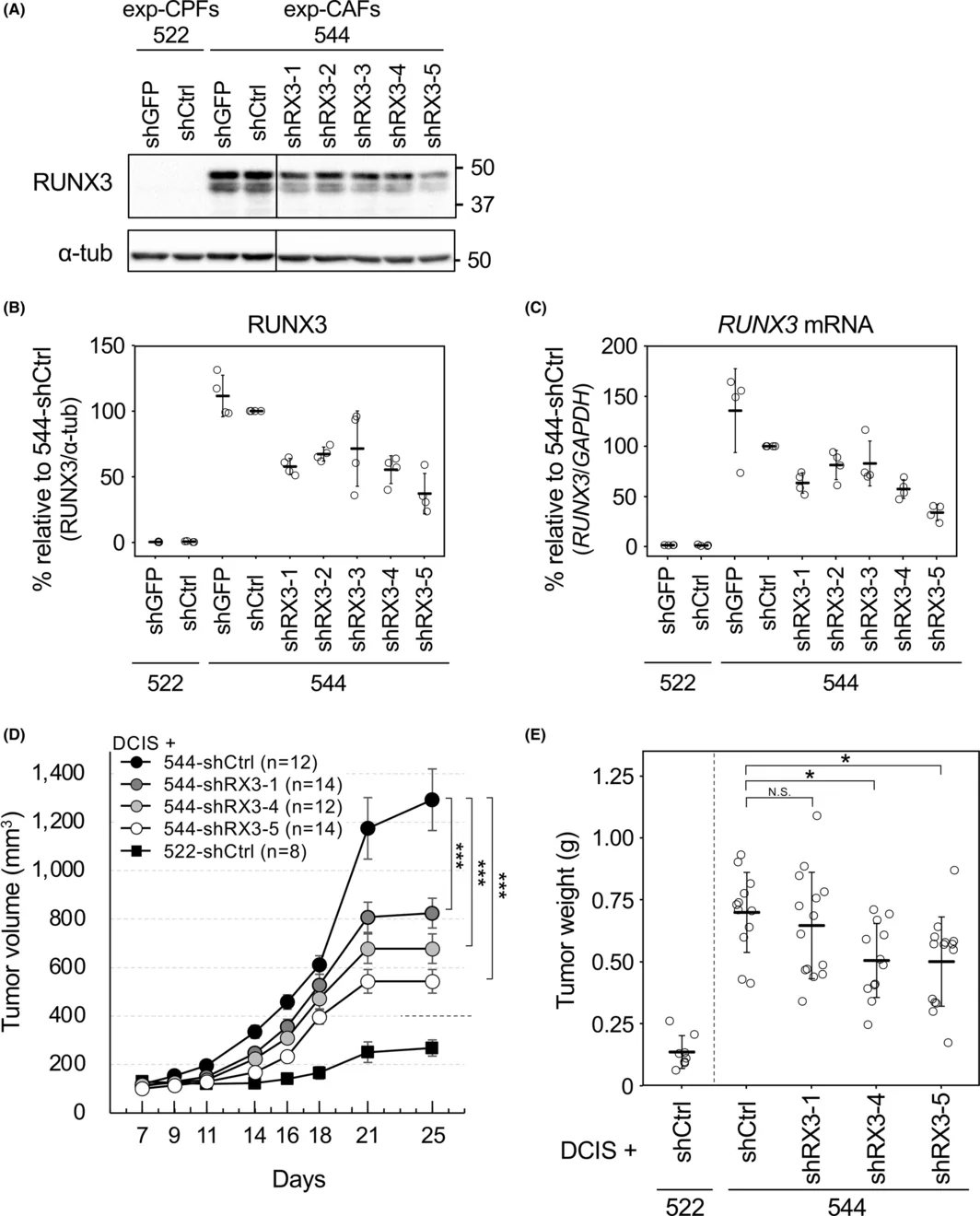
Attenuation of tumor‐promoting ability of exp‐CAF 544 cells by RUNX3 knockdown. (A) Western blot analysis of RUNX3 levels in 522 and 544 cells expressing shCtrl and shGFP and 544 cells expressing five shRNAs targeting to *RUNX3* mRNA (shRX3‐1−5). α‐tub was used as a loading control. (B) Quantitative evaluation of western blot band intensities with four independent experiments. At each experiment, cells were transduced with indicated shRNA expression constructs. Band intensity of RUNX3 was normalized with that of α‐tub, and normalized intensity of RUNX3/α‐tub in 544 cells with shCtrl was expressed as 100 and those in other cells were calculated relative to this at each experiment set. Data with four independent experiments are presented as dot plots showing the means (thick vertical lines) with SD (error bars). (C) RT‐qPCR analysis of *RUNX3* mRNA with four independent experiments. Gene expression levels of *RUNX3* were normalized against those of *GAPDH*. The normalized levels of *RUNX3*/*GAPDH* of 544 cells with shCtrl at each experiment were expressed as 100 and those of other samples were calculated relative to this. The data are presented as in (B). (D) 522 and 544 cells expressing indicated shRNAs were subcutaneously injected with DCIS cells into NOG mice to form tumors. Confirmation of RUNX3 knockdown in the fibroblasts used for injection are shown in Figure S5A. Tumor volumes were measured at indicated days. The numbers of tumors in each group (*n*) are shown in the graph. Statistical analysis was performed between the groups with 544 cells. *** (padj < 0.001) by Dunnett's test. Error bars, SEM. (E) Weight of tumors extracted at 25 days after implantation. Horizontal thick lines represent the means of tumor weight in each group with SD error bars. * (padj < 0.05), N.S. (not statistically significant) by Dunnett's test.

Given the increased RUNX3 expression in human breast CAFs, we next attempted to investigate the involvement of stromal RUNX3 in the promotion of tumor growth. To this end, exp‐CAF 544 cells were transduced with each of five different RUNX3‐targeting shRNA (shRX3‐1−5), control shRNA (nonmammalian sequence shRNA; shCtrl) and GFP‐shRNA (shGFP) constructs. Approximately two weeks after transduction, expression levels of RUNX3 in 544 cells with RUNX3‐targeting shRNAs were decreased between 37% and 71% (the mean values with shRX3‐5 and shRX3‐3, respectively) of those in shCtrl‐expressing 544 cells (Figure 3A,B). Similar reduction was observed in *RUNX3* mRNA levels (Figure 3C). On the other hand, neither protein nor mRNA expression levels of α‐SMA was attenuated by the decrease in RUNX3 expression (Figure S3A−C). It appears that RUNX3 is not required for the maintenance of this myofibroblastic trait in 544 cells.

Then, we selected the three most effective RUNX3‐targeting shRNAs to evaluate the effects of RUNX3 suppression on the exp‐CAF 544 cells' tumor‐promoting ability in vivo (Figure S4). Approximately  two weeks after transduction of shRNA constructs, exp‐CAF 544 cells expressing shRX3‐1, shRX3‐4, shRX3‐5 and shCtrl, and exp‐CPF 522 cells expressing shCtrl were respectively mixed with DCIS breast cancer cells. The mixtures were then implanted subcutaneously into highly immunodeficient NOD/Shi‐*scid*/IL‐2Rγ^null^ (NOG) mice to form xenograft tumors. As shown in Figure 3D, while extensive proliferation of tumors with shCtrl‐expressing exp‐CAF 544 cells (544‐shCtrl) was observed, tumors with exp‐CPF 522 cells expressing shCtrl (522‐shCtrl) grew rather slowly. Twenty‐five days after co‐implantation, averaged volumes of tumors with 544 cells expressing shRX3‐1, shRX3‐4 and shRX3‐5 (544‐shRX3‐1, 544‐shRX3‐4 and 544‐shRX3‐5) were significantly decreased to 64%, 52%, and 42%, respectively, compared to that with 544‐shCtrl (Figure 3D and Figure S5B). Crucially, the weight of resected tumors generated with cancer cells and 544‐shRX3‐4 or 544‐shRX3‐5 showed significant reduction; the mean tumor weight of both groups was 72% of that with 544‐shCtrl (Figure 3E). The mean tumor weight with 544‐shRX3‐1 was lower (92%) than that with 544‐shCtrl without statistical significance.

**FIGURE 4:**
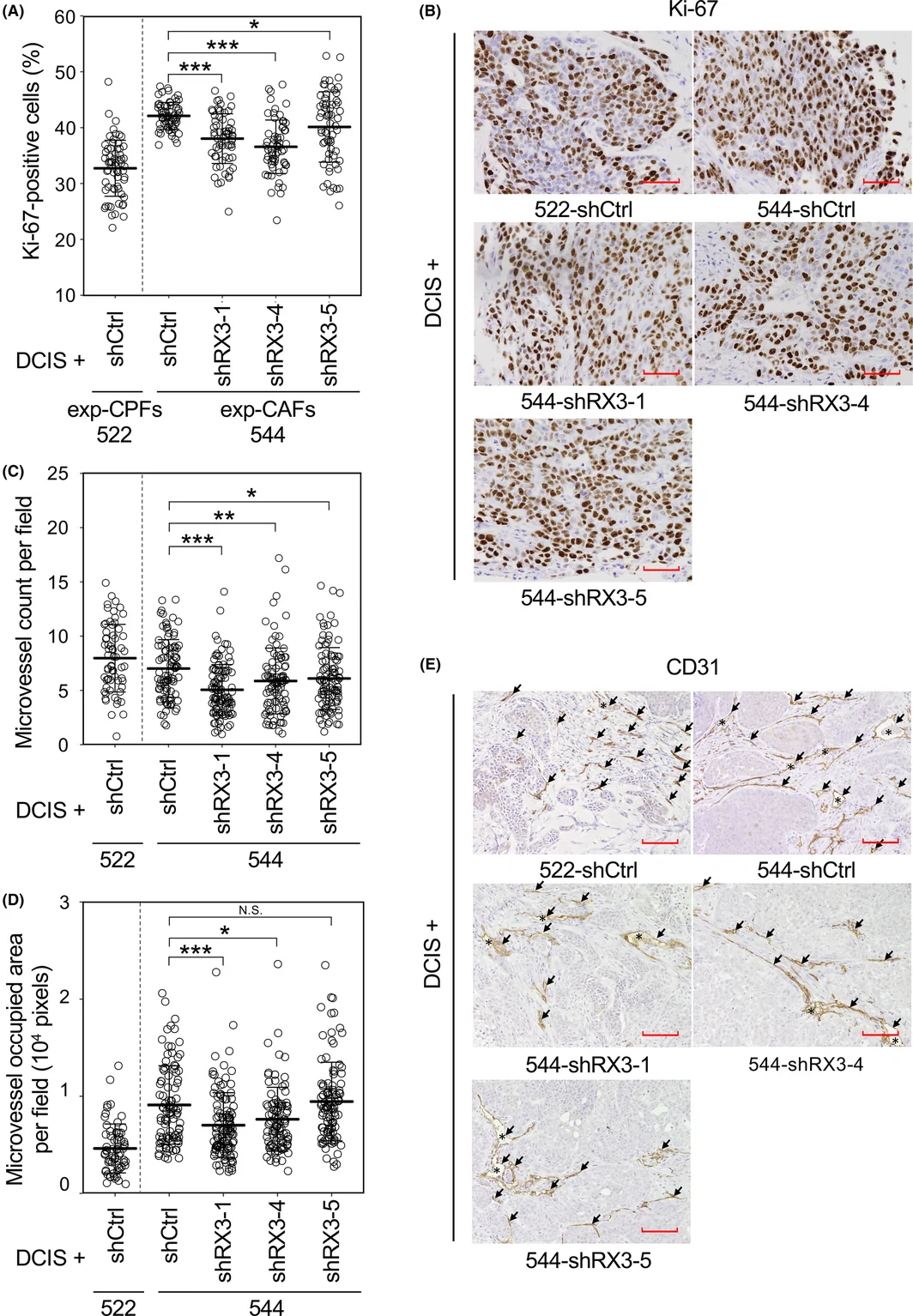
Immunohistochemical evaluation of cell proliferation and microvessel formation in extracted xenograft tumors. (A) Eight fields rich in Ki‐67‐positive cells were chosen from the sections of tumors generated with DCIS cells and 522 or 544 cells expressing indicated shRNAs and the percentage of Ki‐67‐positive cells to Ki‐67‐positive and ‐negative cells were calculated and plotted in the graph. The number of observed fields (*n*) was 64 (the number of xenografts: 8) for all groups. Horizontal thick lines represent the means of each group with SD error bars. *(padj < 0.05), ***(padj < 0.001) by Dunnett's test. (B) Examples of immunohistochemical staining images of xenograft tumor sections with anti‐Ki‐67 antibodies (brown). Hematoxylin staining was also performed (purple). Scale bar in red: 50 μm. (C, D) Eight fields rich in CD31 staining were chosen from each tumor section and the number of CD31‐positive cell‐surrounded areas were counted (C) and CD31‐positive cell‐surrounded areas including CD31‐positive cells were measured as microvessel occupied areas (D). *n* = 64 (8) for 522‐shCtrl; *n* = 96 (12) for 544‐shCtrl; *n* = 112 (14) for 544‐shRX3‐1; *n* = 96 (12) for 544‐shRX3‐4; *n* = 104 (13) for 544‐shRX3‐5. The data are presented as in (A). *(*p* < 0.05), **(*p* < 0.01), *** (*p* < 0.001), N.S. by Steel's test. (E) Examples of immunohistochemical staining images of xenograft tumor sections with anti‐CD31 antibodies. Hematoxylin staining was also performed. Arrows indicate CD31‐positive cells. Asterisks: large microvessels. Scale bar in red: 100 μm.

Since suppression of RUNX3 expression in 544 cells attenuated the xenograft tumor growth in recipient mice, we addressed whether RUNX3 in the exp‐CAFs contributes to co‐injected cancer cell proliferation and to neoangiogenesis in the tumors. Sections prepared from the extracted tumors were stained immunohistochemically with anti‐Ki‐67 and anti‐CD31 antibodies. Ki‐67 is an indicator of cell proliferation and CD31 is a marker of vascular endothelial cells to visualize microvessels. The proportion of Ki‐67‐positive cells was significantly lower in tumor sections generated with 544‐shRX3‐1, ‐shRX3‐4 and ‐shRX3‐5 than that with 544‐shCtrl (Figure 4A,B). Moreover, evaluation of the microvessels with CD31 staining revealed significant reduction of microvessel numbers in the tumor sections with the RUNX3‐knockdown exp‐CAFs than those with the control cells (Figure 4C,E). In addition, microvessel areas are significantly smaller when tumors were formed with 544‐shRX3‐1 or 544‐shRX3‐4, but not with 544‐shRX3‐5 (Figure 4D,E). Overall, the staining results can explain the decreased xenograft tumor growth upon suppression of 544 cells' RUNX3 expression.

### 3.4 Suppression of RUNX3 expression downregulates the expression of *RASL11A* in exp‐CAF 544 cells

**FIGURE 5:**
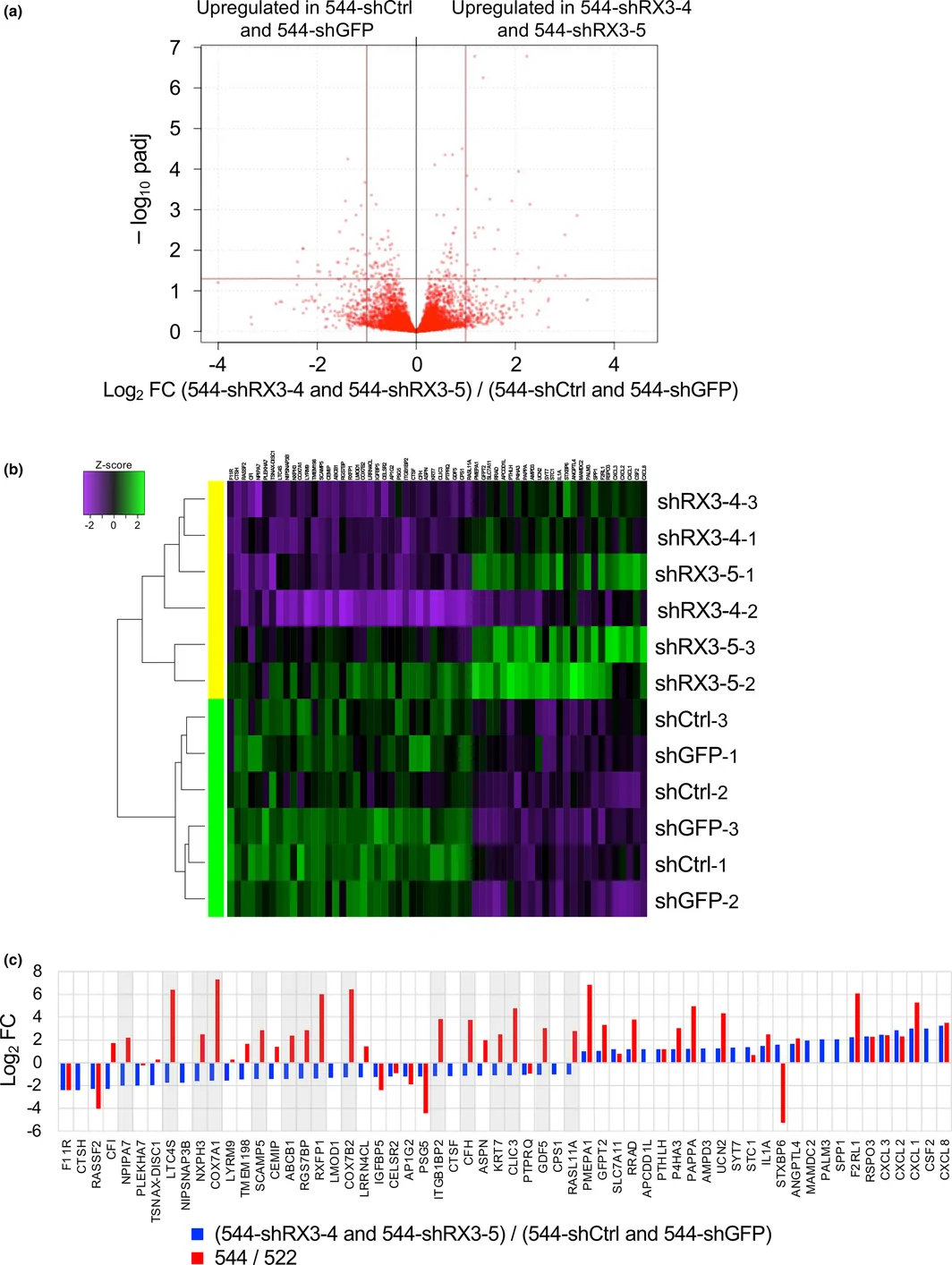
Analysis of gene expression changes upon suppression of RUNX3 expression in exp‐CAF 544 cells. Volcano plots showing differences in the global gene expression between 544 cells with shCtrl and shGFP and those with RUNX3‐targeting shRNAs (shRX3‐4 and ‐5) that significantly reduced tumor weight (Figure 3E). RNA‐seq results with three independent RNA preparations were used. X‐and Y‐axes respectively show the log_2_ FC in the control groups relative to the RUNX3‐knockdown groups and − log_10_ of padj. Log_2_FC of ±1 (X‐axis) and a padj of 0.05 (Y‐axis) are indicated by brown lines. (B) Heatmap presentation of unbiased hierarchical clustering of expression levels of differentially expressed genes (log_2_ FC | > 1; padj <0.05) between 544‐shRX3‐4 and ‐shRX3‐5 and 544‐shCtrl and ‐shGFP. RNA‐seq results with three independent RNA preparations were used. Yellow and pale green horizontal bars at the left indicate 544 cells with and without RUNX3 knockdown, respectively. (C) Bar graph presentation of the differentially expressed genes listed in (B). Blue bars show log_2_ FC of 544‐shRX3‐4 and ‐shRX3‐5 relative to 544‐shCtrl and ‐shGFP and red bars log_2_ FC of 544 cells to 522 cells obtained with DESeq2 analysis. Genes whose expressions are up‐regulated in 544 cells compared to 522 cells and are downregulated upon RUNX3 knockdown in 544 cells are highlighted with gray.

To gain an insight into the molecular mechanism underlying how the decreased RUNX3 expression attenuated tumor‐promoting ability of exp‐CAF 544 cells, we performed RNA‐seq and investigated differentially expressed genes between two groups: 544‐shCtrl and ‐shGFP (544 cells expressing shGFP) and 544‐shRX3‐4 and ‐shRX3‐5, the latter of which significantly reduced the tumor weight (Figure 5A). Expression levels of 60 genes were significantly changed at more than twofolds, of which 25 and 35 genes were up‐ and down‐regulated, respectively, in the RUNX3‐suppressed 544 cells (Figure 5B). shGFP was used as another control shRNA, because comparison of gene expression profiles between 544‐shCtrl and 544‐shGFP showed no gene that satisfies the conditions of significantly differentially expressed genes (log_2_FC | > 1 and adjusted *p* < 0.05 in DESeq2 analysis^27^) except for a single gene.

The five most upregulated genes in the RUNX3‐knockdown group were CXC chemokine ligand 8 (*CXCL8*/*IL‐8*), colony stimulating factor 2 (*CSF2*/*GM‐CSF*), *CXCL1*, *CXCL2*, and *CXCL3* (Figure 5B). In addition, changes in the gene expression profiles were analyzed between 544‐shCtrl and ‐shGFP and 544‐shRX3‐4 and ‐shRX3‐5 using gene set enrichment analysis (GSEA) with Hallmarks gene set^29^, ^30^ (Figure S6). Genes involved in TNFα‐signaling via NFκ‐β, inflammatory response and interferon α/γ response were enriched in the cells with RUNX3 knockdown, which is consistent with the genes found to be upregulated with the DESeq2 analysis. However, when we examined the expression levels of *IL‐8*, *CXCL1*, *CXCL2* and *CXCL3* individually by RT‐qPCR using samples from four independent shRNA knockdown trials, expression patterns of these genes did not appear to correspond with the extent of RUNX3 suppression nor tumor growth attenuation (Figure S7 and Figure 3A−C). Especially, the expression levels of the above genes in 544‐shRX3‐1 appear to be lower than 544‐shCtrl. Also, their expression levels vary considerably among the four knockdown trials with shRX3‐5.

Since RUNX3 is a transcription factor, we speculated that a certain RUNX3‐regulated gene(s) is directly involved in the tumor‐promoting property of exp‐CAF 544 cells. Analysis of RNA‐seq data suggested no significant reduction in the expression levels *SDF‐1* and *TGF‐β* genes (Figure S8) whose upregulations are likely to support the tumor‐promoting property of the exp‐CAFs.^19^, ^20^ Then, we looked into the above 60 genes (Figure 5B) with the following criteria: the gene expression is significantly increased with Log_2_FC >2 in 544 cells (high RUNX3 expression) compared to 522 cells (no RUNX3 expression) and is downregulated in 544 cells upon RUNX3 knockdown as it resulted in decreased tumor growth promotion in vivo (Figure 3E). Fifteen genes, *NPIPA7, LTC4S, NXPH3, COX7A1, SCAMP5, ABCB1, RGS7BP, RXFP1, COX7B2, ITGB1BP2, CFH, KRT7, CLIC3, GDF5*, and *RASL11A*, were met with the criteria (Figure 5C). We then examined the binding of RUNX3 to these genes in published RUNX3 ChIP‐seq data using ChIP‐Atlas.^31^ It was suggested that RUNX3 binds to the exon 1 regions of *RASL11A* gene and an *ABCB1* isoform gene in human blood cells (Figure S9). In addition, the exon 1 regions of these genes were also bound by RUNX3 in mouse blood cells (Figure S9). These data suggest a possibility that *RASL11A* serves as the RUNX3‐downstream gene that mediates CAFs' tumor‐promoting ability.

## 4 DISCUSSION

In this study, we employed an exp‐CAF line which had previously been established from immortalized human mammary fibroblasts by co‐implantation with breast cancer cells in recipient mice. Long‐term in vivo incubation appears to have converted the injected fibroblasts into activated myofibroblasts, exp‐CAF 544 cells.^20^ Because the exp‐CAFs are conferred the potent ability to induce tumor growth, invasion and metastasis and showed enhanced expression of TGF‐β, SDF‐1, and α‐SMA,^20^, ^21^ which are typical CAF signatures, they can be a useful experimental tool to gain a deeper understanding of CAFs' characteristics and their communication with cancer cells in the tumor microenvironment.

We compared gene expression profiles of exp‐CAF 544 and exp‐CPF 522 cells and found that RUNX3 transcription factor is highly expressed in the former, while it was not detected in the latter. Then, we observed significantly more RUNX3‐positive fibroblast‐like cells in cancerous regions than in noncancerous regions in tissue specimens from 10 breast cancer patients. Also, our analysis of a published microarray dataset^28^ suggests a trend of higher levels of *RUNX3* mRNA in primary culture fibroblasts from carcinomas than those from adjacent noncancerous breast tissue from six breast cancer cases. These findings indicate that RUNX3 expression is upregulated in CAFs at least in two independent breast cancer patient cohorts. Because RUNX3 is sometimes functionally inactivated due to cytoplasmic mislocalization in cancer cells,^9^ we examined subcellular localization of RUNX3 in exp‐CAF 544 cells. RUNX3 was detected in the nucleus exclusively, suggesting that this transcription factor could be active in the exp‐CAFs. Nuclear localization of RUNX3 is likely due to TGF‐β autocrine signaling proposed in the exp‐CAFs,^20^ as TGF‐β was shown to induce translocation of RUNX3 from cytoplasm to the nucleus.^13^

Because RUNX3 is abundantly present in exp‐CAF 544 cells, we next investigated whether RUNX3 contributes to their tumor‐promoting ability. To this end, we knocked down RUNX3 expression in the fibroblasts, grafted them with DCIS breast cancer cells under the skin of highly immunodeficient NOG mice and demonstrated that knockdown of RUNX3 in the exp‐CAFs could suppress the growth of tumors formed with the exp‐CAFs and cancer cells. Crucially, among the three shRNAs used for xenograft, two of those most efficiently downregulated RUNX3 expression reduced the tumor weight significantly. This finding supports our view that the tumor‐weight reduction is attributable to RUNX3 downregulation and not from nonspecific effects of shRNA expression. Moreover, Ki‐67 immunohistochemical staining of the resulting tumors suggests that cell proliferation activity was less prominent in the xenograft tumors generated with the exp‐CAFs expressing RUNX3‐targeting shRNAs (shRX3‐1, ‐4 and ‐5) than those expressing negative control shRNA. Regarding neoangiogenesis, microvessel detection with CD31 staining indicated that suppression of RUNX3 expression in the tumor‐promoting fibroblasts affected the microvessel numbers significantly and reduced the microvessel‐occupied areas with two out of three shRNAs. These data imply that the tumor growth is partially influenced by neoangiogenesis under the current experimental setting. Collectively, our xenograft experiment results suggest that RUNX3 expression in CAFs could contribute to the promotion of tumor growth, at least, through stimulating the tumor cell proliferation and blood vessel formation in human breast cancer.

Our tissue microarray analysis of 241 breast cancer patients indicated that the stromal RUNX3‐positive group had a better outcome of survival than the stromal RUNX3‐negative group. A previous work demonstrated that gene expression profiles of tumor stroma microdissected from 53 primary tumors in breast cancer patients are useful for prediction of their clinical outcomes.^16^ Stromal RUNX3 expression was included in the gene set associated with good clinical outcomes.^16^ This is in line with our tissue microarray analysis results. Thus, RUNX3 could be used as a prognosis marker for breast cancer; stromal RUNX3 expression is positively associated with good outcome. Considering the results of xenografts and patient tissue microarray together, it is possible that while overexpression of RUNX3 in CAFs contributes to tumor proliferation, it does not promote metastasis, the major cause of death in breast cancer.^32^ Conversely, when stromal RUNX3 expression is weak or negative, tumor proliferation may be slower but metastasis may occur more frequently. In this study, we did not evaluate metastasis upon RUNX3 knockdown in the exp‐CAFs. This would be a future work to further understand the mechanism of stromal RUNX3 involvement in the tumor malignancy.

RUNX3 knockdown in exp‐CAF 544 cells did not decrease the expression of α‐SMA, a typical marker for myofibroblastic CAFs. Consistently, RUNX3 knockdown did not downregulate the expression levels of *SDF‐1* and *TGF‐β* genes, which were shown to induce α‐SMA expression in the exp‐CAFs.^20^ We previously found that SDF‐1 inhibition suppressed the growth of xenograft tumors formed by the mixture of SDF‐1‐producing CAFs and breast cancer cells^19^ and that two autocrine signaling loops involving SDF‐1 and TGF‐β are cross‐communicating.^20^ Considering the results in the current study and previous findings together, we speculated that attenuation of exp‐CAF 544 cells' tumor‐promoting ability by RUNX3 knockdown is likely dependent on a signaling pathway(s) in which neither SDF‐1 nor TGF‐β is involved. Therefore, we searched for genes whose expression levels show similar patterns to the trend of tumor growth: it is expressed at higher levels in exp‐CAF 544 cells than in exp‐CPF 522 cells and decreased by RUNX3 knockdown in the former. We then sorted the listed genes based on whether their exon 1 regions are suggested to be bound by RUNX3. Analysis of our RNA‐seq data and published RUNX3 ChIP‐seq data suggested that *RASL11A* might play a role in the tumor‐promoting ability of the exp‐CAFs and that it is under the control of RUNX3 at the transcription level. RASL11A is intriguing as it was identified as a member of Ras super‐family with a possibility of being a tumor suppressor.^33^ A later study proposed that RASL11A is a regulator of RNA polymerase I transcription.^34^ Additionally, based on GSEA analysis, the oxidative phosphorylation pathway appears to have been down‐regulated by RUNX3 suppression in the exp‐CAFs (Figure S6). Further studies are required to elucidate whether and how RASL11A and the oxidative phosphorylation pathway influence the tumor‐promoting ability of CAFs.

We observed enhanced gene expression of *IL‐8*/*CXCL8*, *CXCL1*, *CXCL2*, *CXLC3*, and *CSF2* in exp‐CAF 544 cells expressing shRX3‐4 and shRX3‐5, which achieved the most efficient knockdown among the five shRNAs used in this study. However, shRX3‐1, which achieved the third most efficient knockdown, appears to have induced downregulation of the *CXCL* gene expression. Also, shRX3‐5 caused a large variation of their expression between the knockdown trials. Therefore, it is unlikely that these genes are significantly associated with the RUNX3 knockdown‐induced growth suppression of xenograft tumors. Presumably, gene expression of the cytokines is a nonspecific event related to innate immune response to double‐stranded RNAs that were generated from expressed shRNA molecules.^35^

In this study, we found increased RUNX3 expression in exp‐CAF 544 cells and in the stromal fibroblast‐like cells in the cancerous regions in breast cancer patient specimens. Moreover, we demonstrated that the downregulation of RUNX3 expression in the exp‐CAFs suppresses the growth of xenograft tumors generated from co‐implantation of the exp‐CAFs and breast cancer cells in vivo. We propose that RUNX3 can mediate the tumor‐promoting ability of CAFs. More study is needed to understand the relationship between the RUNX3's involvement in the tumor‐promoting ability and the association of this protein with good clinical outcome, from which we could seek for a possibility to focus on RUNX3 as a therapeutic target in the future.

## AUTHOR CONTRIBUTIONS

**Yu Koyama:** Formal analysis (lead); funding acquisition (equal); investigation (lead); validation (equal); writing – original draft (supporting); writing – review and editing (supporting). **Hiroya Okazaki:** Formal analysis (lead); investigation (lead); validation (equal); writing – original draft (supporting); writing – review and editing (supporting). **Yang Shi:** Formal analysis (supporting); investigation (supporting). **Yoshihiro Mezawa:** Investigation (supporting); methodology (supporting). **Zixu Wang:** Investigation (supporting); methodology (supporting). **Mizuki Sakimoto:** Investigation (supporting); methodology (supporting). **Akane Ishizuka:** Investigation (supporting); methodology (supporting). **Yasuhiko Ito:** Investigation (supporting). **Takumi Koyama:** Investigation (supporting). **Yataro Daigo:** Formal analysis (equal); investigation (equal); methodology (equal). **Atsushi Takano:** Formal analysis (supporting); investigation (supporting). **Yohei Miyagi:** Investigation (supporting). **Tomoyuki Yokose:** Investigation (supporting). **Toshinari Yamashita:** Investigation (supporting). **Keisuke Sugahara:** Supervision (supporting). **Okio Hino:** Funding acquisition (supporting); resources (equal); supervision (supporting). **Liying Yang:** Investigation (supporting). **Reo Maruyama:** Investigation (supporting). **Akira Katakura:** Resources (equal); supervision (lead). **Takehiro Yasukawa:** Conceptualization (lead); data curation (equal); formal analysis (lead); investigation (supporting); methodology (lead); project administration (lead); supervision (lead); validation (lead); writing – original draft (lead); writing – review and editing (lead). **Akira Orimo:** Conceptualization (lead); formal analysis (supporting); funding acquisition (lead); investigation (supporting); methodology (lead); resources (equal); supervision (lead); writing – review and editing (supporting).

## FUNDING INFORMATION

This work was supported in part by the research funding from Department of Oral Pathobiological Science and Surgery, Tokyo Dental College, Grant‐in‐Aid for Scientific Research on Innovative Areas from the Japan Society for the Promotion of Science (JSPS KAKENHI Grant Number JP: 25640069, 18K07207, 15K14385 to A. O. and 16H06277 to Y. D., Y. M.), Grant‐in‐Aid for Research Activity Start‐up from JSPS (20K23119 to Y.K.), Grant‐in‐Aid for Early‐Career Scientists from JSPS (22K17217 to Y.K.), Grant‐in‐Aid (S1311011) from the Foundation of Strategic Research Projects in Private Universities from the MEXT, Japan (A. O.) and Juntendo University School of Medicine, Research Institute for Diseases of Old Age (A. O.).

## CONFLICT OF INTEREST STATEMENT

T. Yamashita received Honoraria from Chugai Pharmaceutical, Eisai, Pfizer, Daiichi Sankyo and Eli Lilly.

## ETHICS STATEMENT

Use of FFPE tissue specimens for immunohistochemical analysis was approved by the Juntendo University ethics review board. For tissue microarray analysis using 241 breast cancer patient specimens, individual institutional ethics committees approved this study and use of all clinical materials. Informed consent was obtained. Experiments were carried out in accordance with all guidelines and regulations indicated by the committees and conformed to the provision of the Declaration of Helsinki. Registry and the Registration No. of the study/trial: N/A. Animal experiments were approved by the Animal Research Ethics Committee of the Juntendo University, Faculty of Medicine.

## Supporting information


Data S1.

## Data Availability

Supporting information, including Supplementary Tables S1−S4 and Supplementary Figures S1−S9, is available online. The raw data from RNA‐sequencing experiments in this study are deposited in GSE240453.
