# Supplementary material for: Increased RUNX3 expression mediates tumor‐promoting ability of human breast cancer‐associated fibroblasts
Source: Cancer Med. 2023 Aug 28;12(17):18062–77. doi: 10.1002/cam4.6421 (PMC10523979; doi:10.1002/cam4.6421)
Supplement: Supplementary file 1 — Data S1. [file CAM4-12-18062-s001.pdf]

* Supporting Information *

Increased RUNX3 expression mediates tumor-promoting ability of human breast cancer-associated fibroblasts

Yu Koyama[1,2]†, Hiroya Okazaki[1,2]†, Shi Yang[2,3], Yoshihiro Mezawa[2,3], Zixu Wang[2,3], Mizuki Sakimoto[2], Akane Ishizuka[2,3], Yasuhiko Ito[2], Takumi Koyama[1,2], Yataro Daigo[4,5], Atsushi Takano[4,5], Yohei Miyagi[6], Tomoyuki Yokose[7], Toshinari Yamashita[8], Keisuke Sugahara[1], Okio Hino[2], Liying Yang[9], Reo Maruyama[9], Akira Katakura[1], Takehiro Yasukawa[2,3]*, Akira Orimo[2,3]*

This PDF file contains:

- **Supplementary Tables 1−4 (Tables S1−S4).**
- **Supplementary Figures 1−9 (Figs. S1−S9) and the legends.**

Table S1. Primers for RT-qPCR

Target RNA		Sequence (5′- to -3′)
RUNX3	Forward	TGGCAGGCAATGACGAGAACTACT
	Reverse	TGAACACAGTGATGGTCAGGGTGA
ACTA2	Forward	GTGTGTGACAATGGCTCTGG
	Reverse	TGGTGATGATGCCATGTTCT
GAPDH	Forward	TCAACTACATGGTTTACATGTTC
	Reverse	GATCTCGCTCCTGGAAGAT
IL-8	Forward	ACTGAGAGTGATTGAGAGTGGAC
	Reverse	AACCCTCTGCACCCAGTTTTC
CXCL1	Forward	AGGGAATTCACCCCAAGAAC
	Reverse	ACTATGGGGGATGCAGGATT
CXCL2	Forward	CCCATGGTTAAGAAAATCATCG
	Reverse	CTTCAGGAACAGCCACCAAT
CXCL3	Forward	GACACTGCAGGGAATTCACC
	Reverse	GGTGCTCCCCTTGTTCAGTA

Table S2. Oligonucleotides for generating shRNA expression constructs

shRNA name		Sequence (5′- to -3′)
shRUNX 3-6	Sense	CCGGACCTCGGAACTGAACCCATTCTACTAGTGAATGGGTTCAGTTCCG AGGTTTTTG
	Antisense	AATTCAAAAAACCTCGGAACTGAACCCATTCACTAGTAGAATGGGTTCA GTTCCGAGGT
shRUNX 3-7	Sense	CCGGGTTCAACGACCTTCGCTTCGTTACTAGTACGAAGCGAAGGTCGTT GAACTTTTTG
	Antisense	AATTCAAAAAGTTCAACGACCTTCGCTTCGTACTAGTAACGAAGCGAAG GTCGTTGAAC
shRUNX 3-8	Sense	CCGGATGGCAGGCAATGACGAGAACTTACTAGTAGTTCTCGTCATTGCC TGCCATTTTTTG
	Antisense	AATTCAAAAAATGGCAGGCAATGACGAGAACTACTAGTAAGTTCTCGTC ATTGCCTGCCAT
shRUNX 3-9	Sense	CCGGACCACCTCTACTACGGGACATTACTAGTATGTCCCGTAGTAGAGGT GGTTTTTG
	Antisense	AATTCAAAAAACCACCTCTACTACGGGACATACTAGTAATGTCCCGTAGT AGAGGTGGT
shRUNX 3-10	Sense	CCGGCGAGAACTACTCCGCTGAGCTTACTAGTAGCTCAGCGGAGTAGTT CTCGTTTTTG
	Antisense	AATTCAAAAACGAGAACTACTCCGCTGAGCTACTAGTAAGCTCAGCGG AGTAGTTCTCG

Target sequences in sense oligonucleotides are underlined.

Table S3. Histopathological information of 10 breast cancer patients

No.	Age	Diagnosis	Grade	pT factor	pN factor	ER	PR	HER2
1	61	IDC	2(HG)	2	1a	-	-	1+
2	75	IDC	3(HG)	2	1a	-	-	3+
3	46	LCIS	-	1	0	+	+	2+
4	53	IDC	1(HG)	1	0	+	+	2+
5	68	IDC	1(HG)	1	0	-	-	1+
6	72	ASC	-	4a	2a	-	+	1+
7	72	IDC	3(HG)	2	0	-	-	1+
8	55	IDC	2(HG)	1c	0	+	+	2+
9	44	IDC	2(HG)	1c	0	+	+	2+
10	58	DCIS	2(NG)	1	0	+	+	3+

Abbreviations: IDC: invasive ductal carcinoma, LCIS: lobular carcinoma in situ, ASC: adenosquamous carcinoma, DCIS: ductal carcinoma in situ, HG: histological grade, NG: nuclear grade, ER: estrogen receptor, PR: progesterone receptor, HER2: human epithelial growth factor receptor type 2.

Table S4. Fold Change of RUNX3 gene expression levels between human primary CAFs and counterpart fibroblasts (n=6) with Affymetrix probes in GSE20086 (28).

Affymetrix probe set	$FC_{CAFs/CFs}$
204197_s_at	1.9 **
204198_s_at	1.8 *
234928_x_at	1.1 *

Abbreviations: CAFs, cancer-associated fibroblasts; CFs, counterpart fibroblasts.

*($p < 0.05$), ** ($p < 0.01$) by paired *t*-test.

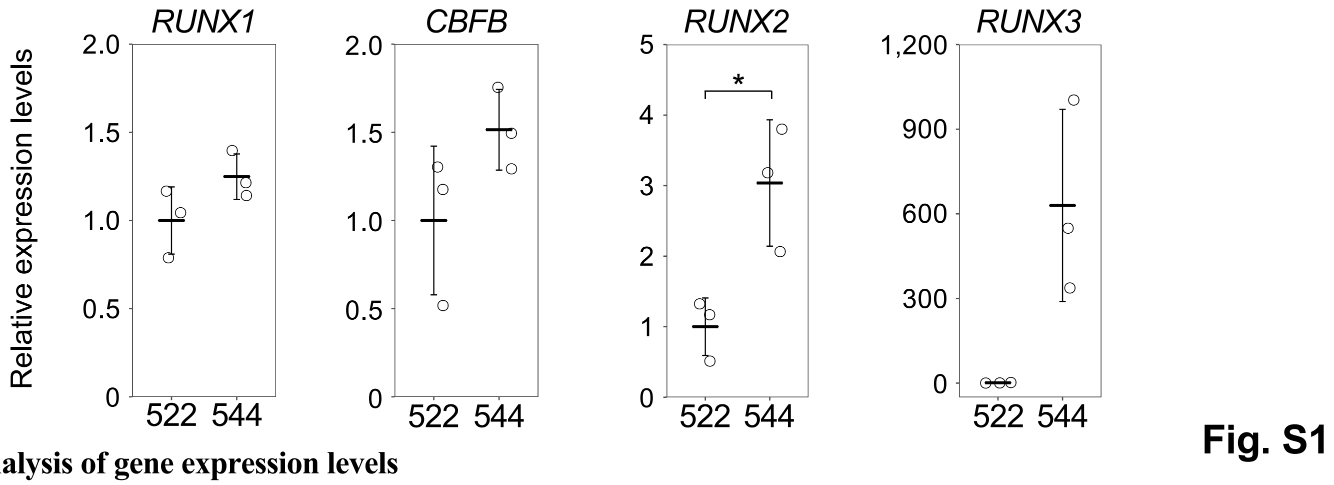

Fig. S1. Analysis of gene expression levels

Relative expression levels of indicated mRNAs in three independent preparations of 522 and 544 cells. Transcription per million (TPM) counts of the indicated genes obtained from RNA-seq results are used. The means of indicated gene expression levels in 522 cells are set as 1 and the relative levels of expression in each sample are shown as dot-plots with the means (thick vertical lines) and SD (error bars). * ($p < 0.05$) by *t*-test.

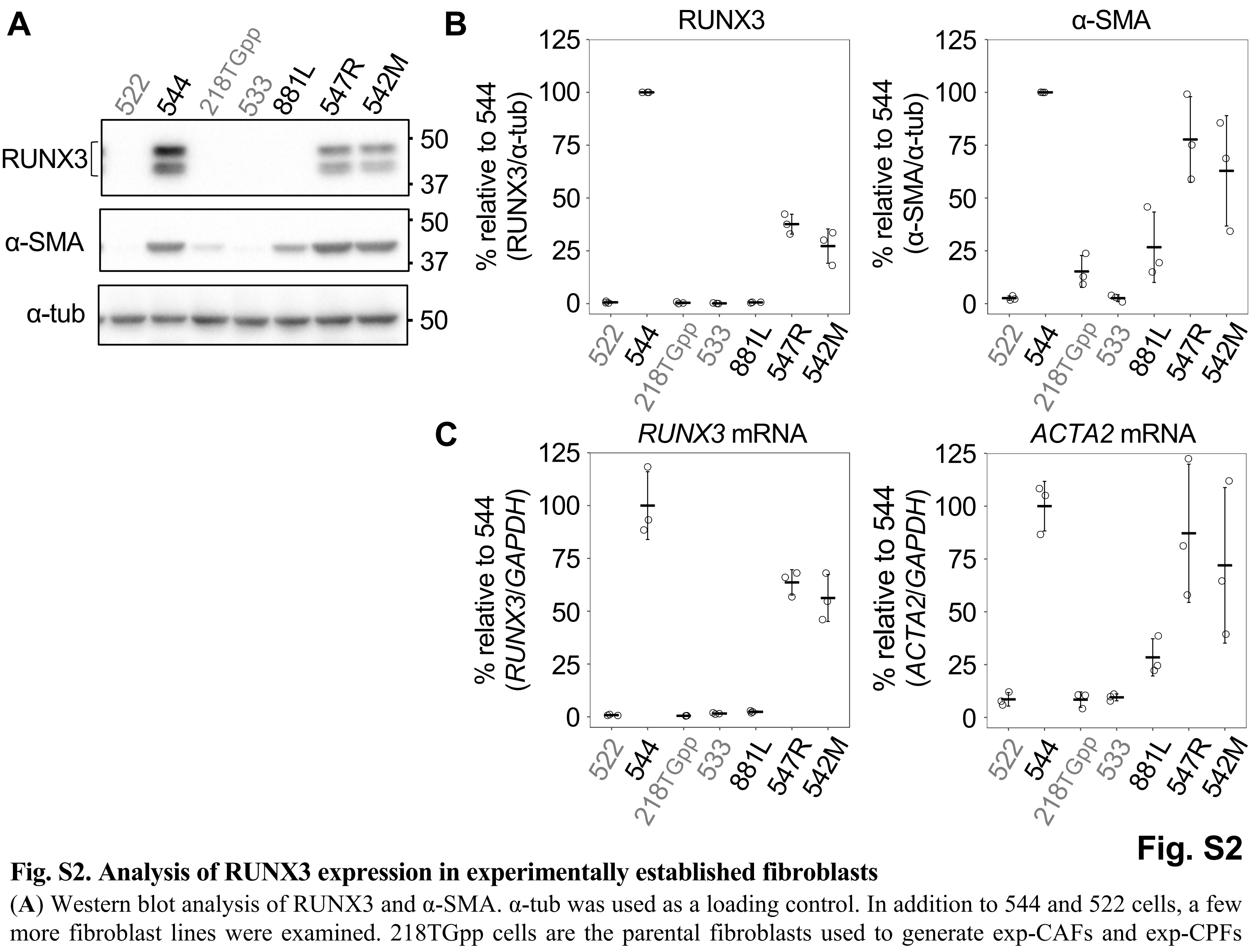

Fig. S2. Analysis of RUNX3 expression in experimentally established fibroblasts

(**A**) Western blot analysis of RUNX3 and α-SMA. α-tub was used as a loading control. In addition to 544 and 522 cells, a few more fibroblast lines were examined. 218TGpp cells are the parental fibroblasts used to generate exp-CAFs and exp-CPFs (20). Like 544 cells, 881L, 547R and 542M cell lines were generated through *in vivo* incubation with breast cancer cells for 85, 170 and 242 days, respectively. 533 cells were generated similarly as 522 cells. 522 and 544 lanes are also shown in Fig. 1B. (**B**) Quantitative evaluation of the western blot band intensities obtained from three independently harvested sample sets. The band intensities of RUNX3 and α-SMA were normalized with that of α-tub. Then, normalized intensities of RUNX3/α-tub and α-SMA/α-tub from 544 cells were expressed as 100 and those from other cell lines were calculated relative to them at each experiment set. Results from three independent preparations are presented as dot plots showing the means (thick vertical lines) with SD (error bars). (**C**) RT-qPCR analysis of *RUNX3* and *ACTA2* mRNAs. Gene expression levels of *RUNX3* and *ACTA2* were normalized against those of *GAPDH*. The normalized levels of *RUNX3*/*GAPDH* and *ACTA2*/*GAPDH* of 544 cells in the three independent preparations were averaged and expressed as 100 and those of all samples were calculated relative to them. The graphs are the average of the results from three independent preparations with SD. 522 and 544 results are also shown Fig. 1C.

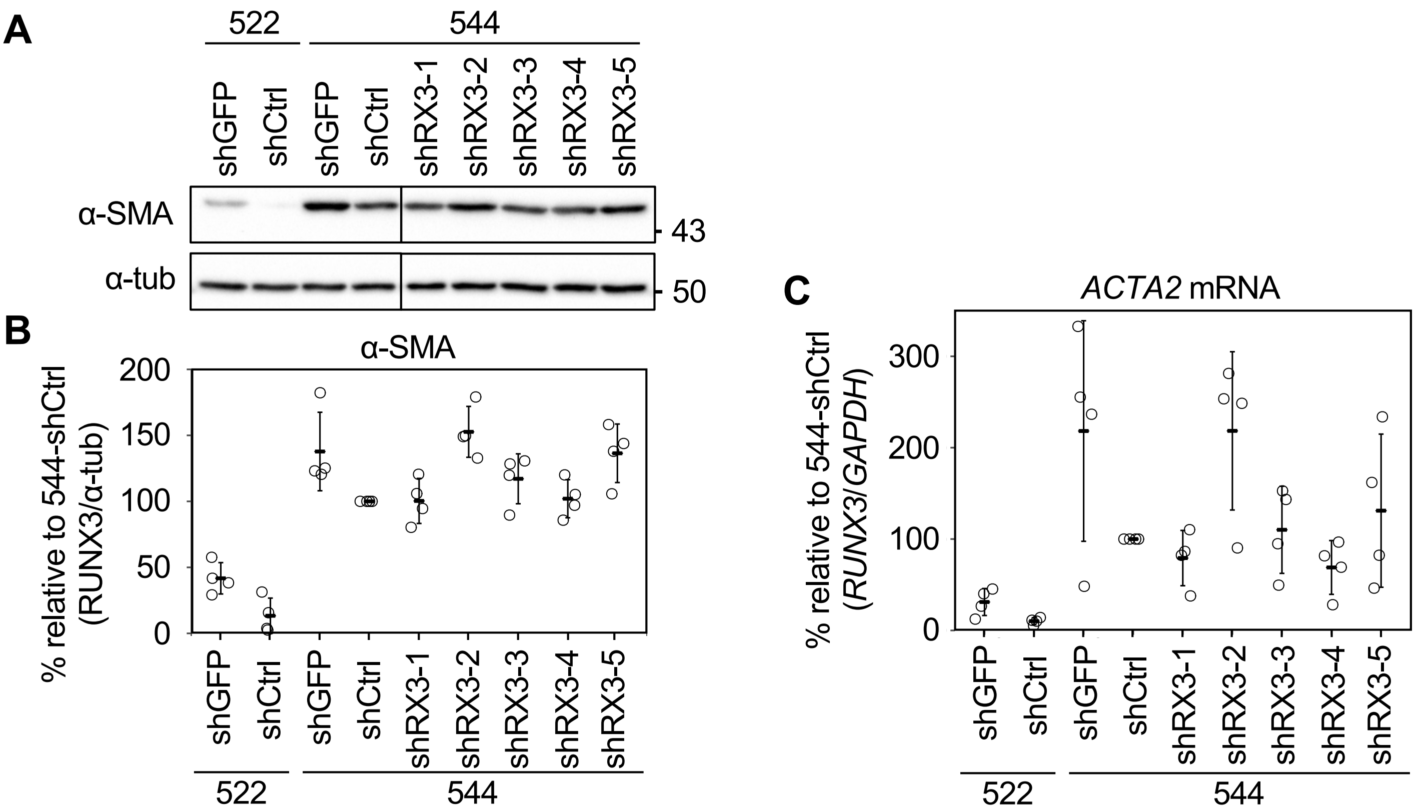

Fig. S3

Fig. S3. Analysis of α-SMA expression upon RUNX3 knockdown

(**A**) Western blot analysis of α-SMA in 522 and 544 cells expressing two control shRNAs (shCtrl and shGFP) and 544 cells expressing five shRNAs targeting to *RUNX3* mRNA (shRX3-1−5). α-tub was used as a loading control. (**B**) Quantitative evaluation of western blot band intensities with four independent experiments. Band intensity of α-SMA was normalized with that of α-tub, and normalized intensity of α-SMA /α-tub in 544 cells with shCtrl was expressed as 100 and those in other cells were calculated relative to this at each experiment set. Data from four independent experiments are presented as dot plots showing the means (thick vertical lines) with SD (error bars). (**C**) RT-qPCR analysis of *ACTA2* mRNA with four independent experiments. Gene expression levels of *ACTA2* were normalized against those of *GAPDH*. The normalized levels of *ACTA2*/*GAPDH* in 544 cells with shCtrl at each experiment were expressed as 100 and those of other samples were calculated relative to this. Data are presented as in (B).

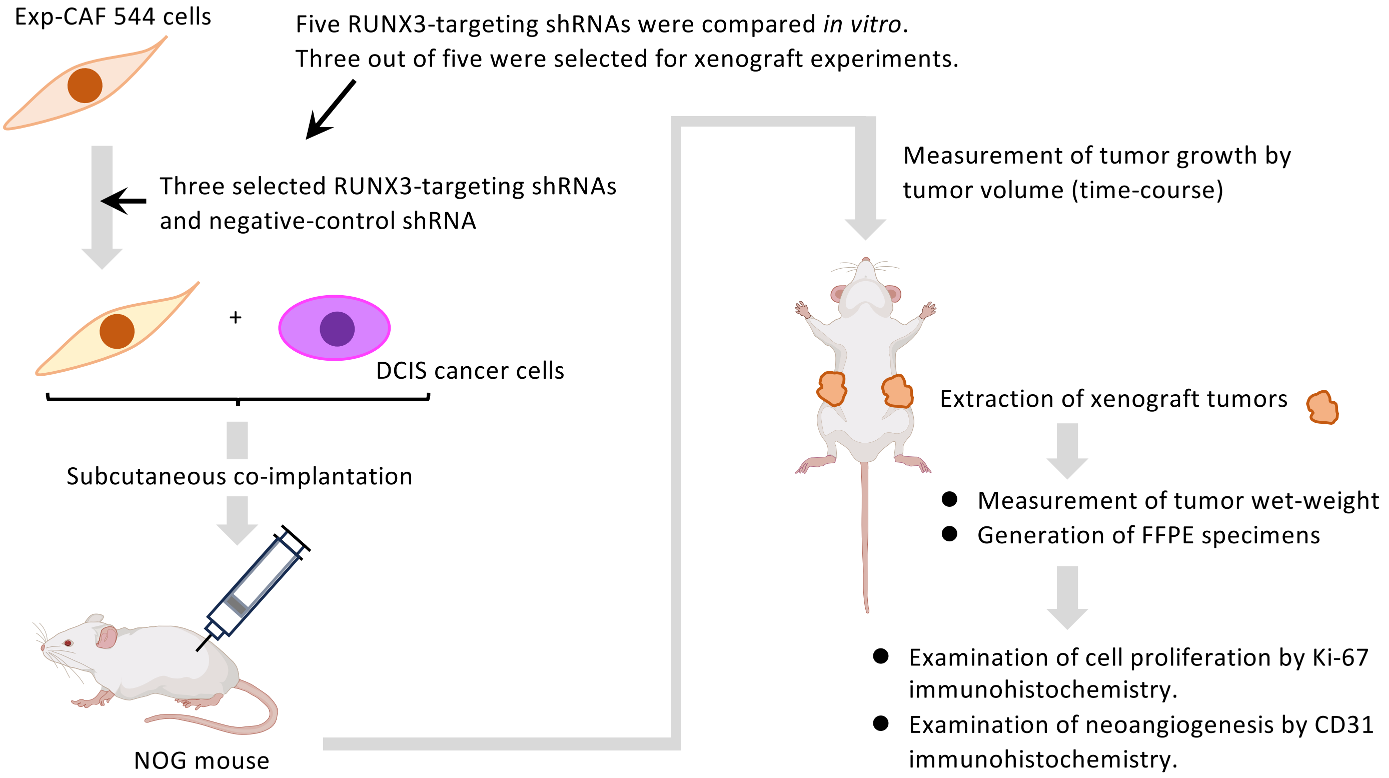

Fig. S4

Fig. S4. Scheme of the xenograft experiment performed in this study

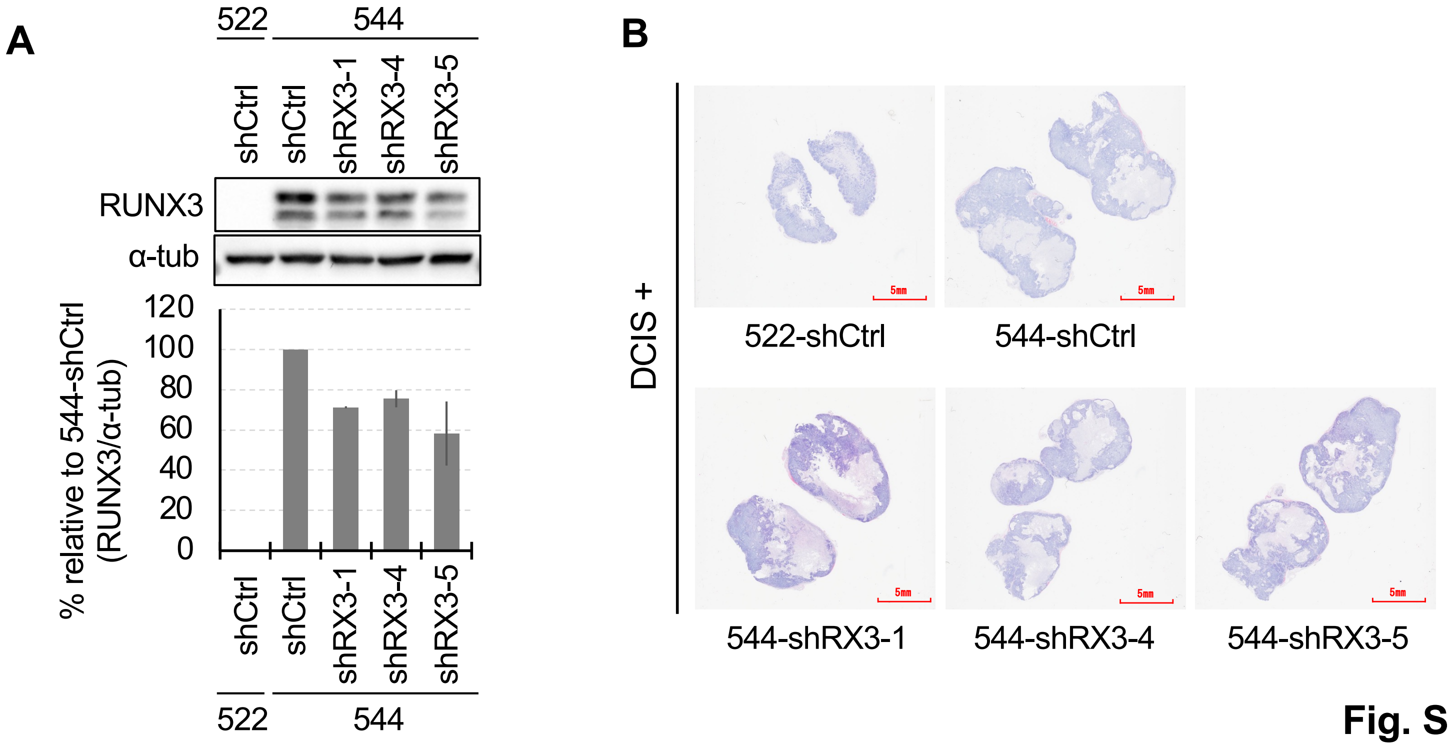

Fig. S5

Fig. S5. Confirmation of RUNX3 knockdown for xenograft

(**A**) Western blot detection of RUNX3 in fibroblasts used for subcutaneous injection with DCIS cells into NOG mice to form xenograft tumors in Fig. 3D and E. The graph shows the quantitative evaluation of RUNX3 band intensity (normalized with α-tub). RUNX3/α-tub in 544-shCtrl was expressed as 100. Western blotting was performed twice with the same sample set and the error bars indicate the error of the means of the technical duplicates. (**B**) Examples of HE-stained xenograft tumor sections. Scale bars; 5 mm.

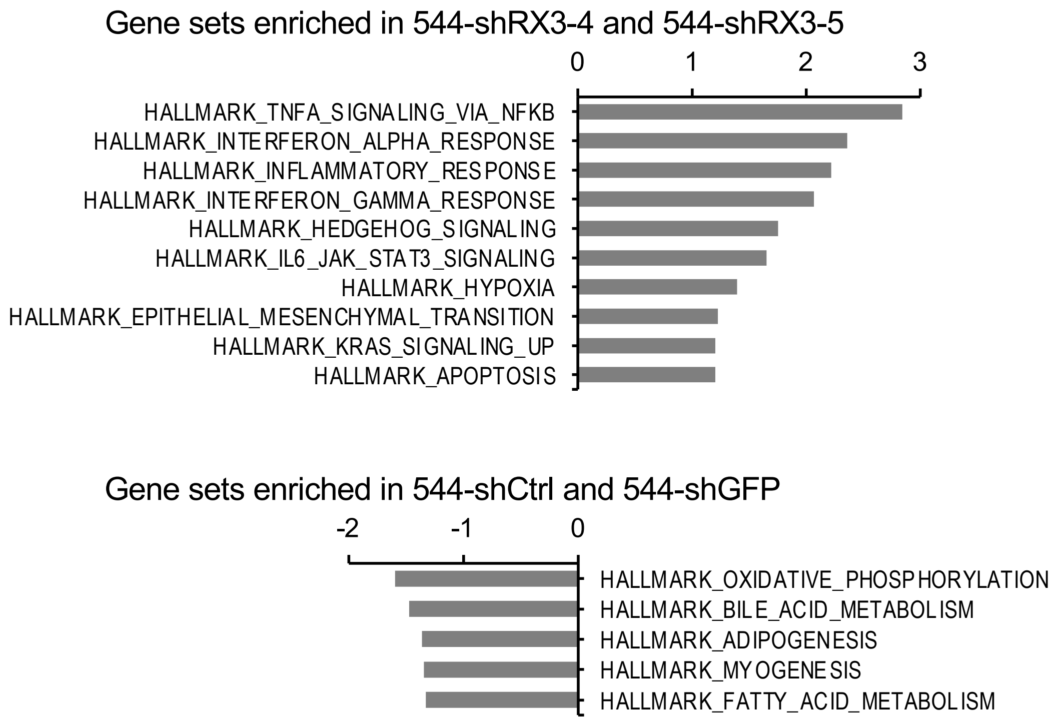

Fig. S6

Fig. S6. Comparison of gene expression profiles between 544 cells with and without RUNX3 knockdown by GSEA

Gene expression profiles between 544-shRX3-4 and -shRX3-5 and 544-shCtrl and -shGFP are compared using GSEA with Hallmarks gene set (30,31).

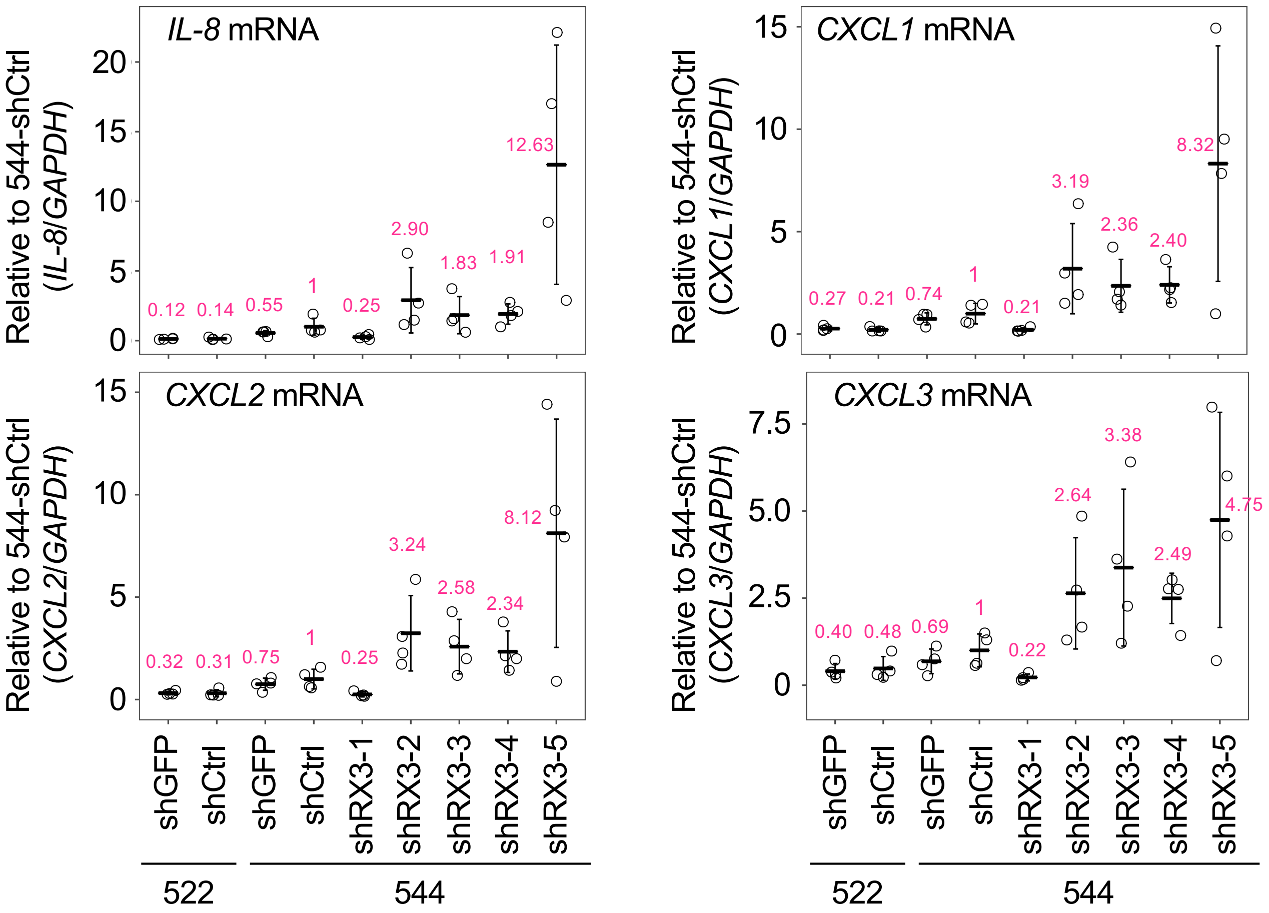

Fig. S7

Fig. S7. Analysis of gene expression levels

RT-qPCR analysis of *IL-8, CXCL1, CXCL2 and CXCL3* mRNA with four independent shRNA transduction experiments. Their gene expression levels were normalized against those of *GAPDH*. Their normalized levels of 544 cells with shCtrl in the four experiments were averaged and expressed as 1 and those of all samples were calculated relative to them. The data are presented as dot plots showing the means (thick vertical lines) with SD (error bars). Mean values (pink color) are indicated.

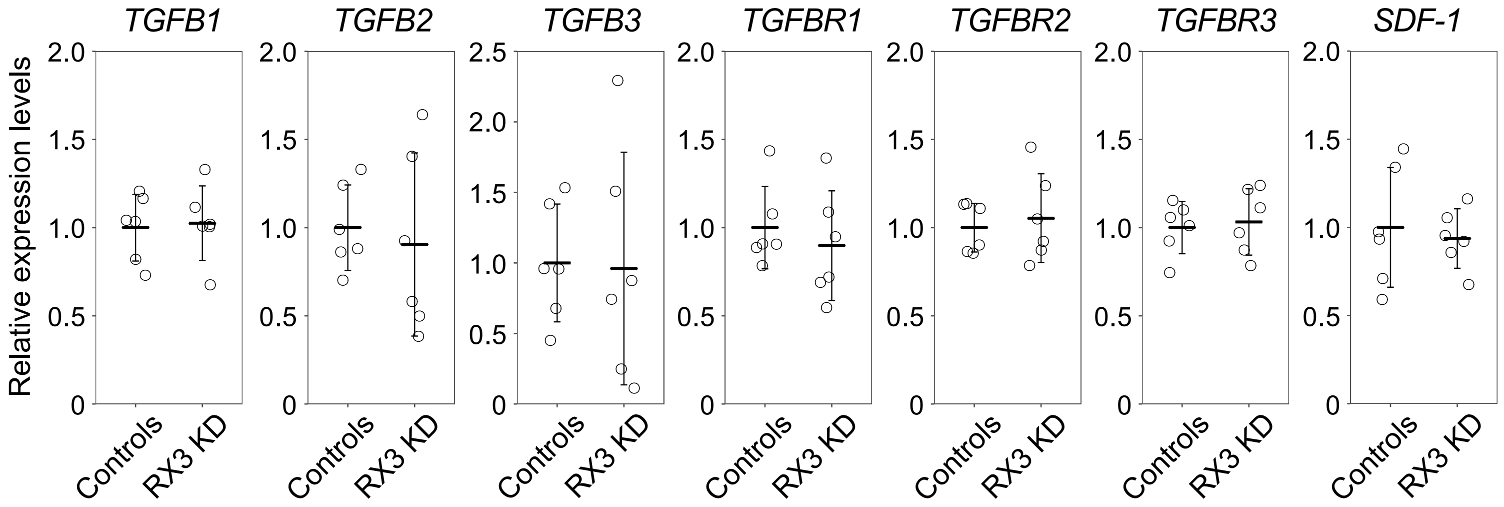

gene	log_2FC	padj	gene	log_2FC	padj
TGFB1	0.115	0.850	*TGFBR2*	0.202	0.620
TGFB2	0.015	0.993	*TGFBR3*	0.203	0.690
TGFB3	-0.501	0.734	*SDF-1*	0.083	0.923
TGFBR1	-0.014	0.988			

Fig. S8

Fig. S8. Relative gene expression levels of *TGFB* genes and *SDF-1*

Relative expression levels of indicated mRNAs in three independent preparations of the control groups (544-shCtrl and -shGFP) and RUNX3-knockdown groups (544-shRX3-4 and -shRX3-5). TPM counts of the indicated genes obtained by RNA-seq were used. The means of the indicated gene expression levels in 522 cells were set as 1 and the relative levels of expression in each sample were shown as dot-plots with the means (thick vertical lines) and SD (error bars). The table shows the values of Log_2 FC and padj of the indicated genes obtained with DESeq2 analysis.

Human *RASL11A*

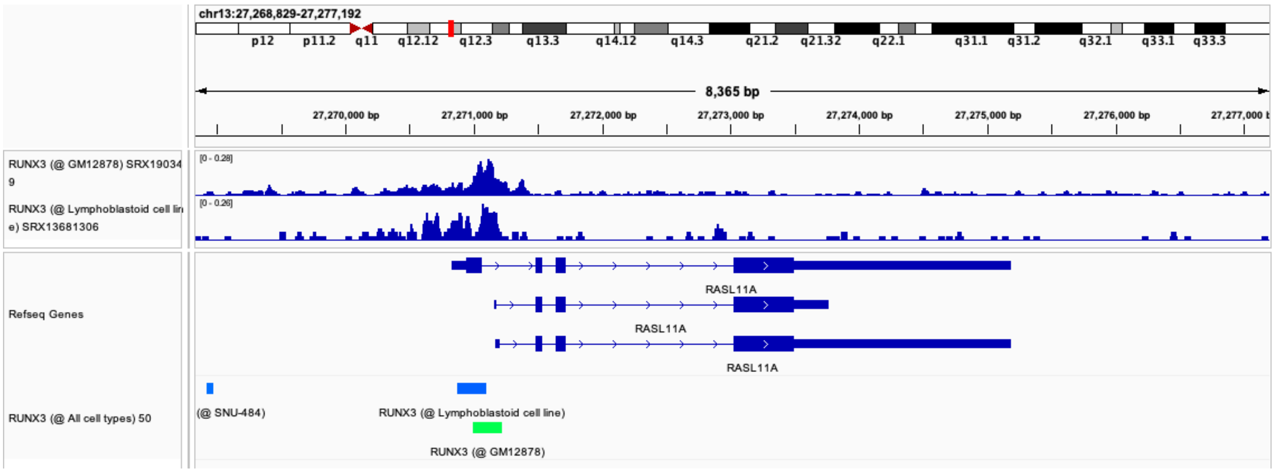

Human *ABCB1*

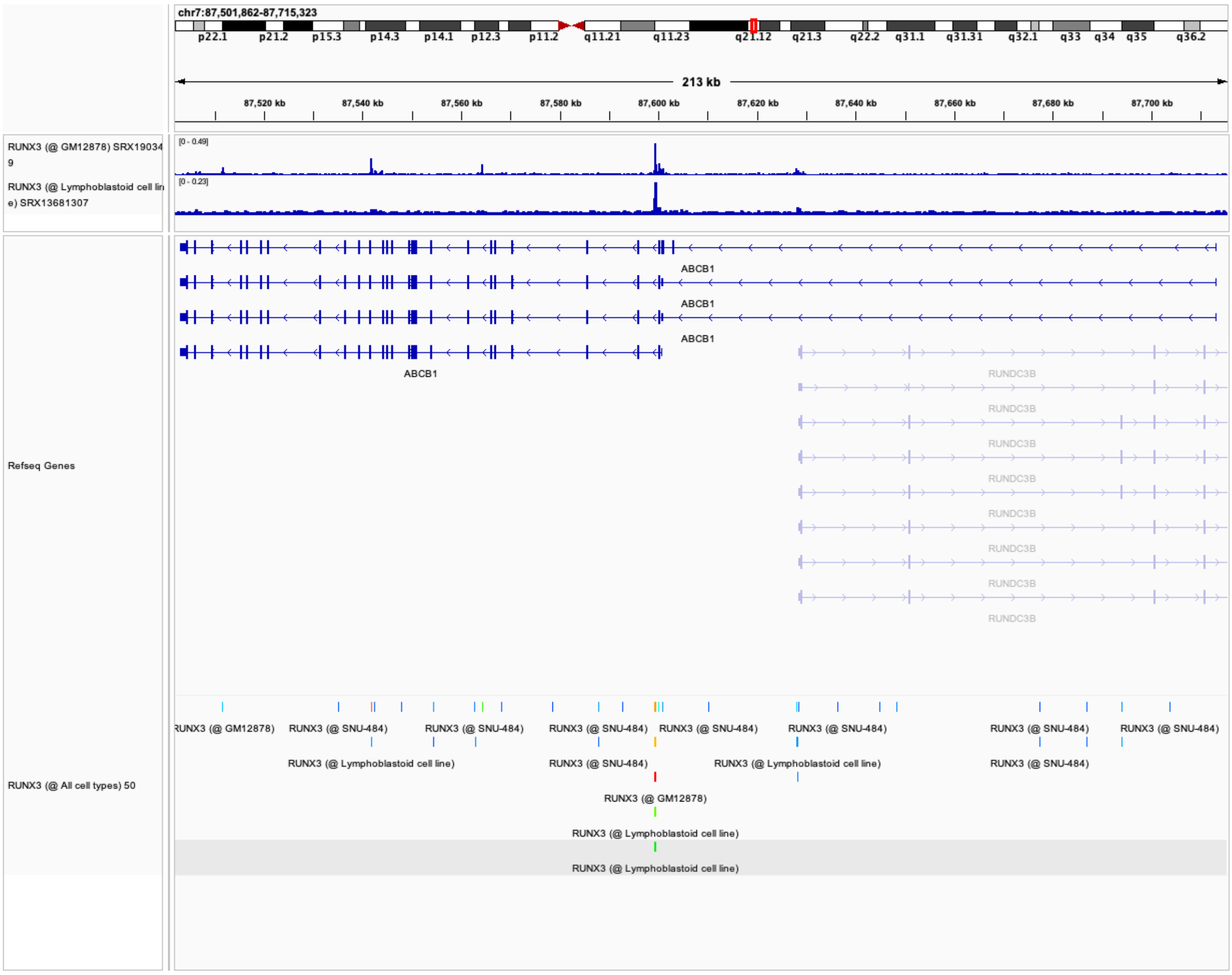

Fig. S9

Mouse *Rasl11a*

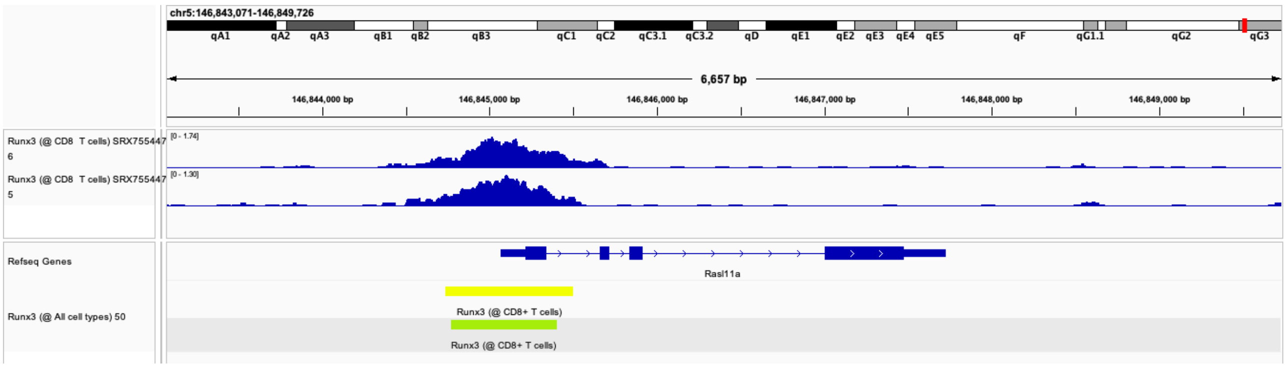

Mouse *Abcb1b*

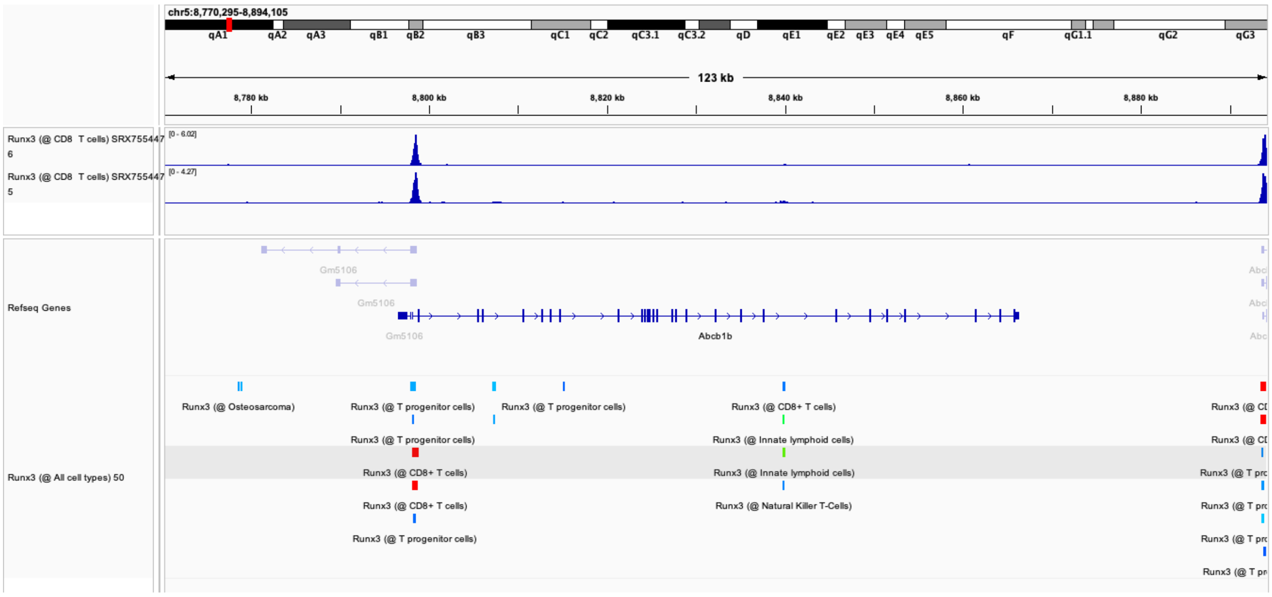

Mouse *Abcb1a*

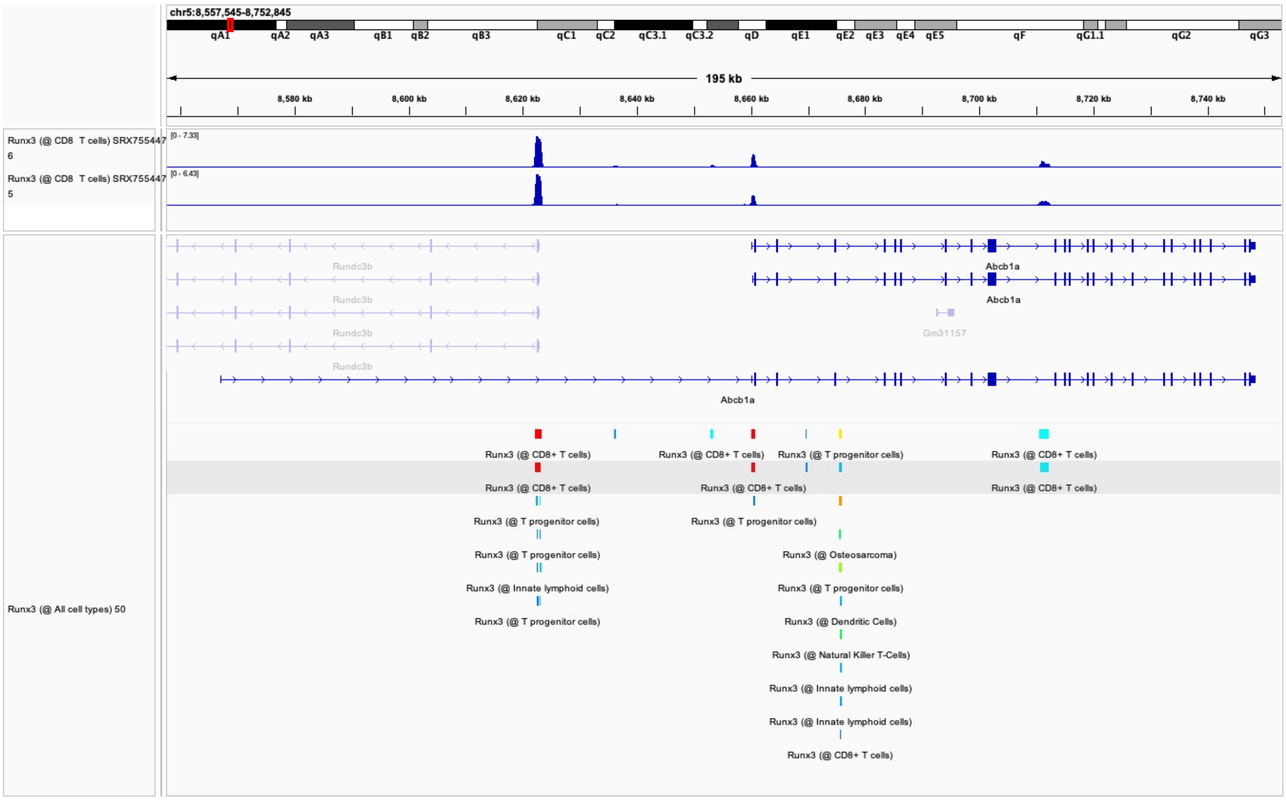

Fig. S9, continued

Fig. S9. RUNX3 binding in published ChIP-seq data

Binding of RUNX3 to the indicated genes were analyzed in published human and mouse RUNX3- ChIP-seq data using ChIP-Atlas (32). Images were generated using ChIP-Atlas.
